# Identification of sites of 2′-O-methylation vulnerability in human ribosomal RNAs by systematic mapping

**DOI:** 10.1038/s41598-017-09734-9

**Published:** 2017-09-13

**Authors:** Sunny Sharma, Virginie Marchand, Yuri Motorin, Denis L. J. Lafontaine

**Affiliations:** 1 RNA Molecular Biology and Center for Microscopy and Molecular Imaging (CMMI), Fonds National de la Recherche (F.R.S./FNRS) and Université Libre de Bruxelles (ULB), BioPark campus Gosselies, Belgium; 2 Next-Generation Sequencing Core Facility, FR3209 BMCT, CNRS-Lorraine University, 9 avenue de la Forêt de Haye, 54505 Vandoeuvre-les-Nancy, France; 3 IMoPA UMR7365 CNRS-UL, BioPole Lorraine University, 9 avenue de la Forêt de Haye, 54505 Vandoeuvre-les-Nancy, France

## Abstract

Ribosomal RNA modifications are important in optimizing ribosome function. Sugar 2′-O-methylation performed by fibrillarin-associated box C/D antisense guide snoRNAs impacts all steps of translation, playing a role in disease etiology (cancer). As it renders adjacent phosphodiester bonds resistant to alkaline treatment, 2′-O-methylation can be monitored qualitatively and quantitatively by applying next-generation sequencing to fragments of randomly cleaved RNA. We remapped all sites of 2′-O-methylation in human rRNAs in two isogenic diploid cell lines, one producing and one not producing the antitumor protein p53. We identified sites naturally modified only partially (confirming the existence in cells of compositionally distinct ribosomes with potentially specialized functions) and sites whose 2′-O-methylation is sensitive to p53. We mapped sites particularly vulnerable to a reduced level of the methyltransferase fibrillarin. The remarkable fact that these are largely sites of natural hypomodification provides initial insights into the mechanism of partial RNA modification. Sites where methylation appeared vulnerable lie peripherally on the 3-D structure of the ribosomal subunits, whereas the numerous modifications present at the core of the subunits, where the functional centers lie, appeared robustly made. We suggest that vulnerable sites of 2′-O-methylation are highly likely to undergo specific regulation during normal and pathological processes.

## Introduction

Ribosomes are essential nanomachines which, in all living cells, convert the information encoded in messenger RNAs into proteins. At the functional core of the ribosome is ribosomal RNA (rRNA), a ribozyme catalyzing the critical steps of decoding and amino acid polymerization^1^. Ribosomal RNA is extensively modified by base isomerization and by covalent addition of chemical groups to nucleotides^2^. RNA modifications extend the topological repertoire of RNAs, stabilizing rRNA structural elements at the core of the ribosomal subunits^3^. Covalent modifications include ribose and base methylation, base acetylation, and base aminocarboxypropylation^4^. Base isomerization consists in the conversion of uridines into pseudouridines. With around fifty of each on yeast ribosomes and 100 of each on human ribosomes, 2′-O-methylations and pseudouridines are by far the most abundant rRNA modifications^2^.

Recent research has revealed the existence of highly specific ribosomal diseases and of specialized ribosomes that might have distinct translational capacities^5^. Ribosomopathies are cancer predisposition syndromes caused by ribosome biogenesis dysfunction resulting from a mutation in either a ribosome assembly factor or a ribosomal protein^6^. There are ribosomes in every cell of our body, but the effects of reduced ribosome production or altered ribosome quality depend on the tissue and cell type^7^. Understanding how this cell/tissue specificity originates is a major challenge in the field. There is increasing evidence that cells produce heterogeneous populations of ribosomes that may differ in composition and translational capacity^8^. Specialized ribosomes may play important roles in normal processes such as embryonic development and in diseases such as cancer^9, 10^. The best demonstration of the coexistence of distinct ribosomes in cells is partial modification, i.e. modification at specific positions on some but not all ribosomes (e.g. refs 11–15). With hundreds of positions on ribosomal RNAs that can either be specifically modified or not, the combinatorial potential to produce diverse ribosomes is immense. Ribosomal RNA modification is thus the prime cause of ribosome diversity^5^.

Ribosomal RNA modifications optimize ribosome function at every step of translation: they influence the mode of translation initiation (cap-dependent versus internal ribosomal entry site dependent initiation), reading frame maintenance, and stop codon recognition^16–21^. RNA modifications may impact ribosomal structure locally^17, 18^, affecting intrinsic properties of the ribosome such as its tRNA and mRNA binding capacity^21^. Changes in the rRNA modification profile therefore can profoundly remodel the translational programs of the cells, with severe pathophysiological consequences^22, 23^. Unsurprisingly, rRNA modifications appear tightly connected to important biological processes such as cell differentiation^24^ and embryonic development^25^, and also to severe disease, in particular cancer^26, 27^.

Recently, deep-sequencing-based strategies have been developed to map RNA modifications at the transcriptome-wide level. These strategies rely on the ability of RNA modifications to be detected by antibodies, to promote specific reverse transcriptase ‘drop off’, to confer specific resistance to RNase digestion, or to induce specific cleavage of the RNA chain when subjected to particular chemical treatments (reviewed in refs 28–30).

‘RiboMethSeq’ allows systematic mapping of 2′-O-methylation sites by deep sequencing on the basis of differential RNA cleavage: phosphodiester bonds directly downstream of 2′-O-methylated residues are selectively more resistant to mild alkaline treatment than those adjacent to unmodified residues^14, 15^. RiboMethSeq has been used successfully to remap all known sites of rRNA 2′-O-methylation in budding yeast^14, 15^ and in human cells^13^ and to identify novel sites. Human rRNA methylation sites have also been mapped by another deep-sequencing-based technique relying on specific reverse-transcriptase drop-off at sites of 2′-O-methylation, stimulated by low nucleotides concentrations^31, 32^.

Ribosomal RNA 2′-O-methylations are installed essentially by the methyltransferase fibrillarin, which is literally ‘carried’ to the sites of modification by specific antisense guide box C/D snoRNAs^33^. Besides its function in RNA modification, fibrillarin is an essential building block of maturing precursor ribosomal subunits and is required as such for essential pre-rRNA processing reactions leading to synthesis of the small ribosomal subunit rRNA^34, 35^. Fibrillarin has also been implicated in rDNA histone methylation^36^. Considering the essential roles of RNA modification in optimizing translation and their involvement in normal and pathological processes, it is essential both to establish their full repertoire and to identify those positions which are liable to undergo specific regulation.

In this work, using RiboMethSeq, we have established the complete repertoire of 2′-O-methylation sites on human ribosomal RNAs in two isogenic diploid cell lines, one producing and the other not producing the antitumor protein p53. We have identified sites of substoichiometric modification, sites whose 2′-O-methylation is sensitive to the presence of p53, and sites whose 2′-O-methylation is particularly affected by a reduction in the intracellular fibrillarin concentration. Sites of partial rRNA 2′-O-methylation provide *bona fide* evidence of the coexistence of compositionally distinct ribosomes in cells. Vulnerable sites of rRNA 2′-O-methylation are prime candidates for specific regulation during normal and pathophysiological processes. Their identification offers a rational framework for future research on ribosomopathies.

## Results

### Systematic mapping of 2′-O-methylation sites on human ribosomal rRNAs

For systematic mapping of 2′-O-methylation sites on human ribosomal RNAs, total RNA was extracted from HCT116 cells and subjected to RiboMethSeq, a deep-sequencing-based strategy relying on differential sensitivity of methylated residues to alkaline treatment^15^. Briefly, total RNA was fragmented by mild alkaline treatment, and the cleaved RNA fragments were used to generate sequencing libraries which were processed by HiSeq 1000^15^. Statistically, RNA cleavage occurs at every position of the rRNA chain, except those located downstream of 2′-O-methylated residues.

We chose to work with HCT116 cells because they are diploid and available as a pair of isogenic sister lines: one that produces p53 (HCT116 p53 +/+) and one that does not (HCT116 p53 −/−)^37^. This makes it possible to test directly for an effect of p53 on the fibrillarin and 2′-O-methylation levels. We systematically used both of these cell lines in our RiboMethSeq analyses.

For each cell line, 14–27 million reads were aligned on reference rDNA sequences, providing extensive coverage, as previously described in yeast^15^. Altogether, our RiboMethSeq analysis identified 106 positions on mature rRNAs: 39 sites on 18S rRNA, 65 sites on 28S rRNA, and 2 sites on 5.8S rRNA (Table 1). No modification was detected on 5S rRNA. For each detected position, a methylation score was computed, reflecting the fraction of rRNA molecules modified in the cell population of ribosomes (Table 1

**Table 1:** Distribution and extent of modification of 2′-O methylation sites on human ribosomal RNAs.

18S	Residue type	snoRNA guide (SNORD)	HCT116 p53 + /+	HCT116 p53 −/−
27	Am	27	0.88	0.83
99	Am	57	0.83	0.78
116	Um	42A, 42B	0.80	0.72
121	Um	4A, 4B	0.92	0.89
159	Am	45A, 45C	0.87	0.79
166	Am	44	0.79	0.75
172	Um	45A, 45B	0.83	0.78
174	Cm	45C	0.83	0.78
428	Um	68	0.8	0.72
436	Gm	100	0.89	0.9
462	Cm	14, 14A, 14B, 14C, 14D, 14E	0.85	0.84
468	Am	83A	0.8	0.76
484	Am	16	0.73	**0.62**
509	Gm	11, 11B	0.87	0.86
512	Am	70	0.75	**0.65**
517	Cm	56, 56B	0.91	0.89
576	Am	93	0.95	0.96
590	Am	62A, 62B	0.78	0.71
601	Gm	85, 103A, 103B	0.81	0.77
627	Um	65	0.88	0.86
644	Gm	54	0.92	0.87
668	Am	36, 36A, 36B	0.84	0.79
683	Gm	19, 19B	0.95	0.86
797	Cm	ZL107	0.73	**0.66**
799	Um	105, 105B	0.91	0.86
*867*	*Gm*	*98*	***0.64***	***0.64***
1031	Am	59A, 59B	0.91	0.88
*1272*	*Cm*	*66*	***0.35***	***0.32***
1288	Um	110	0.85	0.8
1326	Ψm	33	0.84	0.83
1328	Gm	32A, 32B	0.84	0.81
1383	Am	30	0.93	0.88
1391	Cm	28	0.9	0.88
1442	Um	61	0.85	0.79
1447	Gm	127	**0.16**	**0.07**
1490	Gm	25	0.94	0.94
1678	Am	82	0.90	0.88
1703	Cm	43	0.94	0.91
1804	Um	20	0.91	0.90
**28S**	**Residue type**	**snoRNA guide (SNORD)**	**HCT116 p53 +/+**	**HCT116 p53 −/−**
398 (389)	Am	26	0.89	0.86
400 (391)	Am	81	0.92	0.89
*1316 (1303)*	*Gm*	*21*	***0.63***	***0.54***
1326 (1313)	Am	18, 18A, 18B, 18C	0.79	0.67
1340 (1327)	Cm	104	0.69	**0.64**
1522 (1509)	Gm	2	0.83	0.82
1524 (1511)	Am	32A, 32B, 51	0.92	0.83
1534 (1521)	Am	77, 80	0.94	0.92
1625 (1612)	Gm	80	0.96	0.95
1760 (1748)	Gm	73A	0.89	0.87
1871 (1858)	Am	38A, 38B	0.90	0.84
*1881 (1868)*	*Cm*	*48*	***0.47***	***0.28***
2351 (2338)	Cm	24	0.86	0.79
2363 (2350)	Am	76	**0.60**	**0.56**
2364 (2351)	Gm	/	0.91	0.88
**28S**	**Residue type**	**snoRNA guide (SNORD)**	**HCT116 p53 +/+**	**HCT116 p53 −/−**
2365 (2352)	Cm	24	n.d.	n.d.
*2401 (2388)*	*Am*	*68*	***0.69***	***0.54***
2415 (2402)	Um	143, 144	**0.52**	**0.36**
2422 (2409)	Cm	5	0.82	0.78
2424 (2411)	Gm	6	0.91	0.89
2787 (2774)	Am	99	0.8	0.75
2804 (2791)	Cm	55	0.9	0.88
2815 (2802)	Am	95	0.83	0.77
2824 (2811)	Cm	95	0.68	**0.58**
2837 (2824)	Um	34	0.89	0.87
2861 (2848)	Cm	50A, 50B	0.83	0.8
2876 (2863)	Gm	50A, 50B	0.84	0.82
3701 (3680)	Cm	88, 88A, 88B, 88C	0.88	0.82
3718 (3697)	Am	37	0.88	0.85
3724 (3703)	Am	36C	0.94	0.91
3744 (3723)	Gm	87	0.71	**0.55**
3760 (3739)	Am	46	0.89	0.86
3785 (3764)	Am	15A, 15B	0.90	0.82
3792 (3771)	Gm	15A	0.98	0.97
3808 (3787)	Cm	10	0.9	0.89
3818 (3797)	Ψm	17, snoRA48	0.95	0.92
3825 (3804)	Am	30	0.92	0.88
3830 (3809)	Am	79	0.75	**0.64**
3841 (3820)	Cm	74	0.91	0.9
*3867 (3846)*	*Am*	*92*	*0.82*	*0.83*
*3869 (3848)*	*Cm*	*53, 53_92*	*0.87*	*0.81*
3887 (3866)	Cm	47	0.91	0.89
3899 (3878)	Gm	12, 12B	0.90	0.89
3925 (3904)	Um	52	0.73	**0.61**
3944 (3923)	Gm	111, 111B	0.70	**0.57**
4042 (4020)	Gm	102	0.74	**0.61**
4054 (4032)	Cm	75	0.92	0.89
4196 (4166)	Gm	31	0.94	0.93
4227 (4197)	Um	/	0.88	0.85
4228 (4198)	Gm	58A, 58B, 58C	0.89	0.86
4306 (4276)	Um	41	0.79	0.78
4370 (4340)	Gm	60	0.92	0.91
4392 (4362)	Gm	1A, 1B, 1C	0.89	0.86
4456 (4426)	Cm	49A, 49B	0.74	0.71
4494 (4464)	Gm	69	0.90	0.81
4498 (4468)	Um	62A, 62B	0.93	0.93
4499 (4469)	Gm	made by SPB1 in yeast cells	0.75	0.75
4523 (4493)	Am	29	0.86	0.80
4536 (4506)	Cm	35A, 35B	0.95	0.94
4571 (4541)	Am	63	0.8	0.66
4590 (4560)	Am	119	**0.62**	**0.51**
4618 (4588)	Gm	91A, 91B	**0.64**	**0.45**
4620 (4590)	Um	72	0.67	**0.57**
4623 (4593)	Gm	78	0.91	0.88
4637 (4607)	Gm	121A, 121B	0.68	**0.58**
**5.8S**	**Residue type**	**snoRNA guide (SNORD)**	**HCT116 p53 +/+**	**HCT116 p53 −/−**
14	Um	71	0.73	0.71
75	Gm	96A, 96B	0.89*	0.83

Positions of the 2′-O-modified nucleotides identified by RiboMethSeq on HCT116 cells in this work. For 28S rRNA, the revised sequence numbering, taking into account rDNA sequencing errors, is used^38^. The old nomenclature is shown between brackets for reference to previous studies. The chemical nature of the modified residue, the predicted antisense snoRNA guides, and the methylation score (ranging from 0.0 to 1.0), established in both HCT116 p53 +/+ and HCT116 p53 −/− cells by RiboMethSeq are shown. Sites predicted to be hypomethylated are highlighted in bold (cut-off: 0.65). In italics, seven positions identified as fractionally modified in Krogh *et al*. (2016) on the basis of RiboMethSeq and massspec evidence and showing overall good agreement with our data. The data shown in this table correspond to samples treated with SCR for 1 day (see Table S3 for complete dataset). n.d., not determined. In our work, and by comparison to a recent work (Krogh *et al*., 2016), the methylation scores for 5.8S were determined experimentally. * indicates a position in the 5.8S rRNA that was found to be 92% methylated by LC-MS in ref. 48. For this position, we obtained a score of 0.89, which is highly consistent.

).

**Figure 1:**
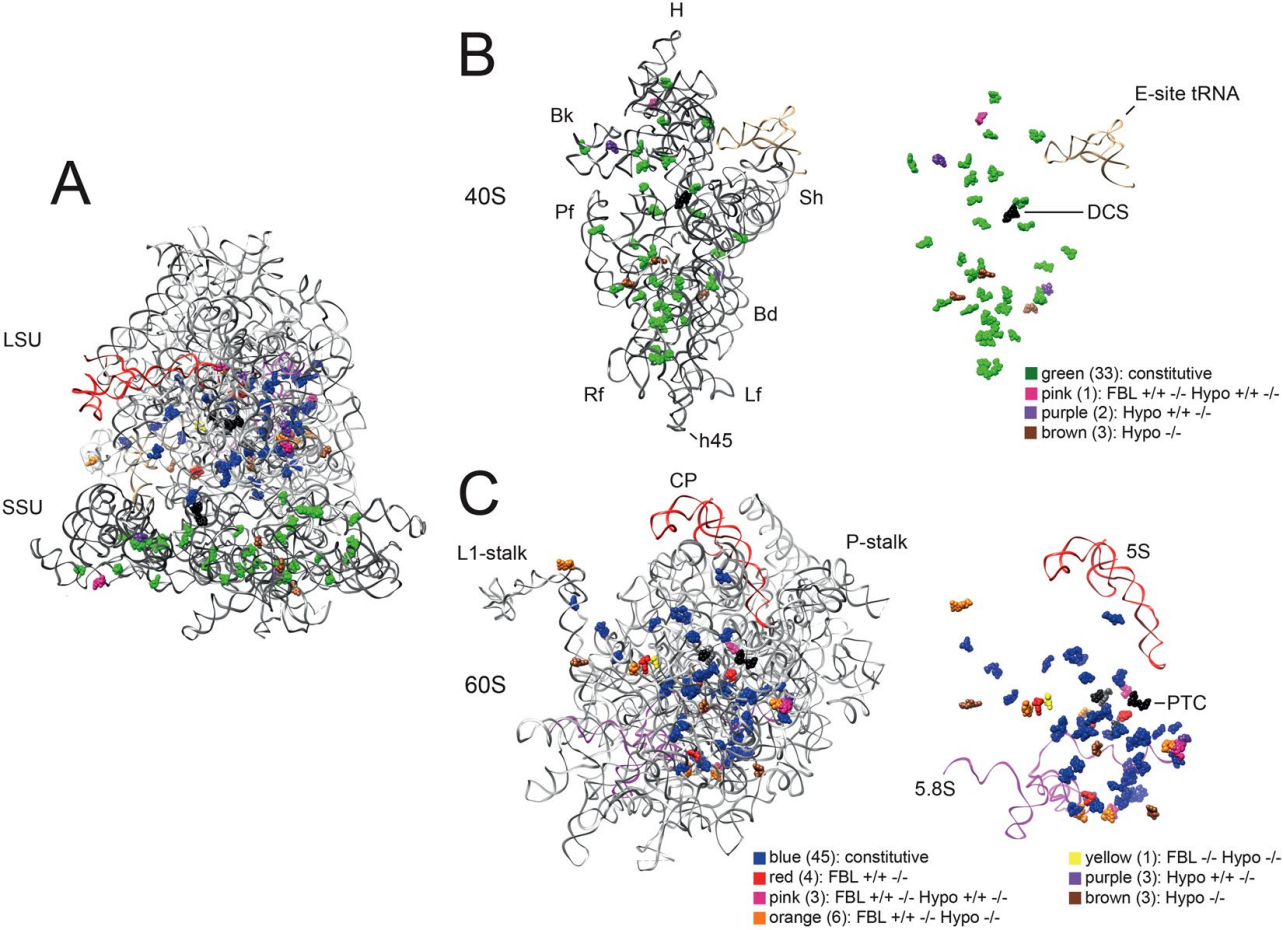
Distribution of the 106 ribose-methylated residues on 3-D models of human ribosomal subunits. (**A**) Entire ribosome: solvent view. The models of the subunits are based on PDB entry 4UG0^47^. The four rRNAs are shown as ribbons (18S, dark grey; 28S, light gray; 5S, red; 5.8S, purple) with the modifications as spheres. The color code used for the modifications is described in panels B and C. SSU, small subunit; LSU, large subunit. (**B**) Small subunit (40S): interface view. Left, 18S rRNA shown as a dark grey ribbon with the modifications as spheres. Right, simplified view showing only the modifications and the E-site tRNA for orientation. The decoding site (DCS) is highlighted in black. Small ribosomal subunit morphological features are indicated: Bk, beak; H, head; Sh, shoulder; Bd, body; Lf, left foot; Rf, right foot; and h45, helix 45. Each modification is color-coded according to the categories identified in this work. (**C**) Large subunit (60S): interface view. Legend as in panel B. The peptidyl transferase center (PTC) residues are highlighted in black. Two PTC residues are 2′-O-methylated (shown in grey). CP, central protuberance.

Each modification site identified was mapped to the revised secondary structures of the rRNAs (Supplementary Figures 1–2) and to the latest 3-D models of human ribosomal subunits (Fig. 1). A major improvement in the revised secondary structures is that they fully depict the eukaryote-specific expansion segments (ES)^38^. Analysis of the 2-D structures revealed broad distribution of modifications over the conserved rRNA elements (Supplementary Figures 1–2), the eukaryote-specific ES being largely devoid of modifications. Analysis of the 3-D subunit models revealed different situations for the small and large ribosomal subunits (Fig. 1). On 40S, the modifications appeared broadly distributed throughout the subunit, including the periphery. They did not appear to concentrate particularly around the decoding site (DCS), but more so in the lower part of the body, in the immediate vicinity of the right foot (Fig. 1B). On 60S, modifications were found to concentrate heavily at the core of the subunit, being largely clustered around the peptidyl transferase center (PTC). They were strikingly scarce at the subunit periphery (Fig. 1C).

### Identification of sites of partial and p53-sensitive 2′-O-methylation

Previous work has demonstrated that RiboMethSeq is quantitative, and that the methylation score determined by this procedure is a reliable measure of the level of RNA modification^13, 15^. In particular, this score has been found to correlate well with the level of methylation determined by LC-MS/MS (see e.g. ref. 15).

For the vast majority of positions identified here, the methylation score was found to be high (Table 1), indicating that most rRNA molecules in cells are modified. Close inspection of the data, however, revealed several sites with low methylation scores on both ribosomal subunits, indicating that only a fraction of the rRNA molecules are modified at these positions (Fig. 1 and Table 1).

On the basis of previous work where a correlation between RiboMethSeq and LC-MS/MS was established for several modification sites (see e.g. ref. 15), we considered a score of 0.65 to be a reasonable threshold for classifying a modification as substoichiometric. Using this threshold, we unveiled six putative sites of hypomodification on the 18S rRNA and sixteen on the 25S rRNA (Table 1). Of these, nine sites were found to qualify as hypomodified in both HCT116 p53 +/+ and HCT116 p53 −/−, while the other thirtheen showed below-threshold scores in HCT116 p53 −/− only. No sites appeared less methylated in HCT116 p53 +/+than in HCT116 p53 −/−.

These data both confirm the existence of partial rRNA modification and, most importantly, show that at some sites, methylation is particularly sensitive to the absence of functional p53 (Table 1). The existence of sites of hypomodification provides *bona fide* evidences of the existence of heterogeneous populations of ribosomes in cells.

### Identification of sites of methylation particularly vulnerable to fibrillarin depletion

At each site, methylation is carried out by an individual snoRNP which must access its substrate residue within a particular timeframe during ribosome biogenesis and within a particular structural neighborhood on the precursor ribosomal subunit. On this basis we reasoned that different sites should be subject to different temporal and structural constraints and be differentially affected by fibrillarin depletion. If so, future research on ribosomopathies is likely to benefit from identifying modification sites which are particularly vulnerable to fibrillarin depletion.

**Figure 2:**
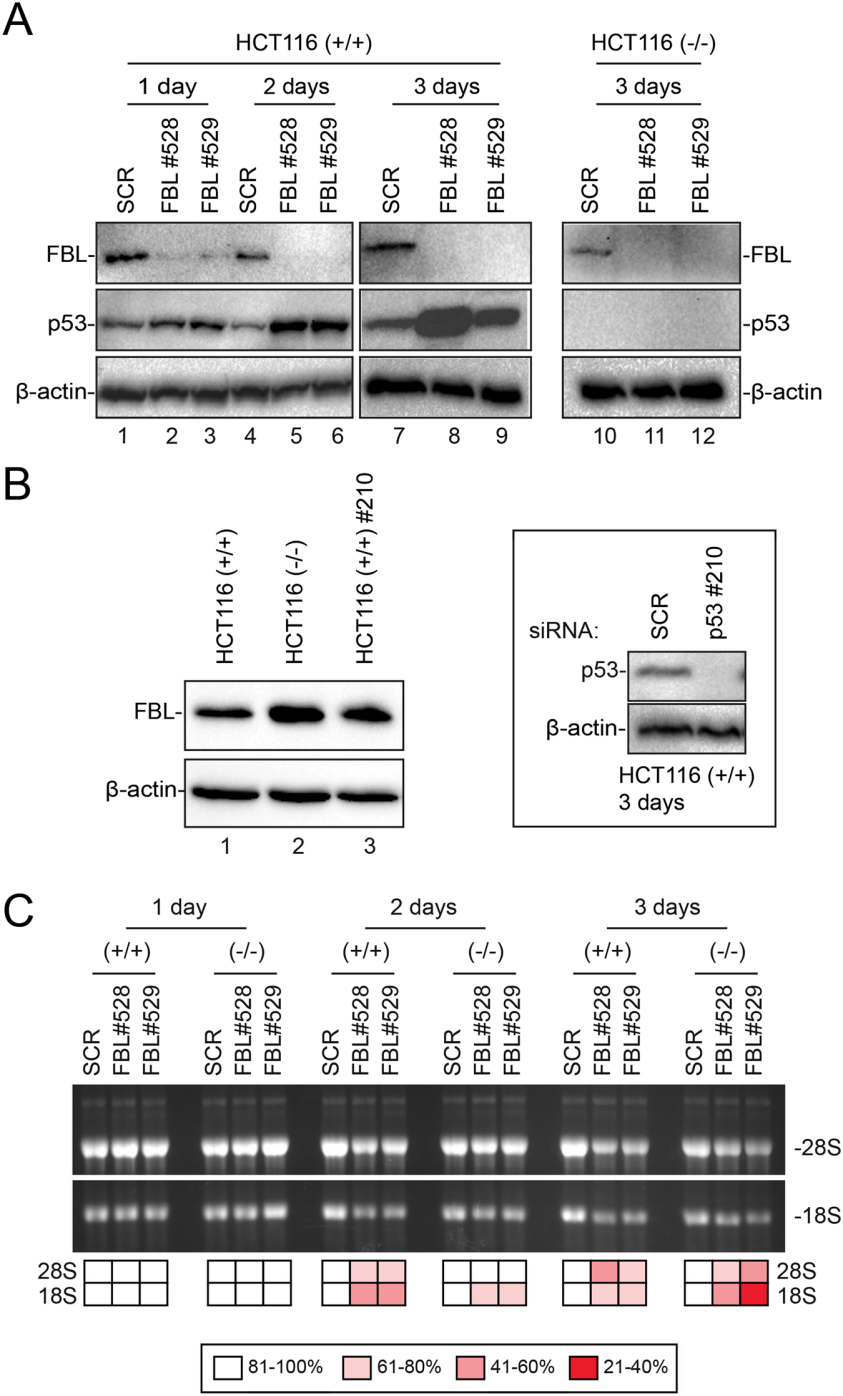
Effects of progressive fibrillarin depletion on ribosome biogenesis and p53 steady-state accumulation. (**A**) Fibrillarin depletion elicits an antitumoral p53-dependent nucleolar stress response. Total protein extracted from HCT116 p53 +/+ and HCT116 p53 −/− cells treated with an siRNA against fibrillarin (#528 or #529) for 1, 2, or 3 days, or with a non-targeting control siRNA (SCR, scramble) analyzed by western blotting with antibodies against fibrillarin (FBL), p53, or β-actin (loading control). Uncropped blots are shown in Supplementary Figure 8. (B) Fibrillarin expression is influenced by p53. Total protein extracted from HCT116 p53 +/+cells, from HCT116 p53 −/− cells, and from HCT116 p53 +/+cells treated with an siRNA against p53 (#210) for 3 days, analyzed by western blotting with antibodies against the indicated proteins (FBL and β-actin). Inset, p53-siRNA-mediated depletion in HCT116 p53 +/+cells is highly efficient. Total protein extracted from HCT116 p53 +/+cells treated for 3 days with an siRNA targeting p53 (#210) or with a non-targeting control siRNA (SCR). The blot was probed with antibodies against the indicated proteins (p53 and β-actin). Uncropped blots are shown in Supplementary Figure 8. (C) Fibrillarin is required for mature rRNA accumulation. HCT116 p53 +/+ and HCT116 p53 −/− cells were depleted of fibrillarin for 1, 2, or 3 days with a specific siRNA (#528 or #529) or with a non-targeting control siRNA (SCR, scramble). Total RNA was extracted and resolved on denaturing gels and mature rRNAs were visualized by ethidium bromide staining. Densitometric quantification of the signals is shown. rRNA levels in cells depleted of fibrillarin are normalized with respect to the levels observed in cells treated with a non-targeting silencer (SCR). The uncropped gel is shown in Supplementary Figure 9. Note that the effects of Fibrillarin depletion on pre-rRNA processing and p53 steady-state accumulation were observed independently at least three times, confirming for the processing data our previous observations^35^ (see Www.RibosomeSynthesis.Com).

To assess systematically the impact of fibrillarin depletion on ribose methylation at individual sites on rRNA, we depleted cells of fibrillarin progressively in a 3-day time course experiment (Fig. 2). In practice, HCT116 cells were transfected with siRNAs targeting FBL mRNAs, and the level of fibrillarin was monitored by western blotting after 1, 2, and 3 days of depletion. Depletion was carried out separately with two different siRNAs (#528 and #529). As a control, cells were treated with a non-targeting control siRNA (scramble, SCR). Depletion was done in both HCT116 p53 +/+ and HCT116 p53 −/− in order to assess the possible influence of p53 on the fibrillarin level and on 2′-O-methylation. Importantly, we established by differential fluorescent DNA staining of live and dead cells that the percentage of viable cells was similar at each time point analyzed (Supplementary Figure 3).

Western-blot analysis established that siRNA-mediated fibrillarin depletion was both effective and progressive: low residual levels of the protein were detected after 24 hours of depletion and no fibrillarin was detected from 48 h onward (Fig. 2A). That depletion was gradual was confirmed at the mRNA levels by an RT-qPCR (data not shown). The two cell lines showed similar depletion efficiency (compare lanes+/+ and−/−).

According to a recent report, p53 can transcriptionally repress fibrillarin synthesis by binding to the DNA sequence encoding its first intron^27^. We therefore examined whether and to what extent the fibrillarin level might be influenced by p53 in our cell lines (Fig. 2B). On the one hand, we directly compared fibrillarin levels in HCT116 p53 +/+ and HCT116 p53 −/− cells (Fig. 2B, lanes 1–2). On the other hand, we depleted HCT116 p53 +/+cells of p53 by treating them with a specific siRNA (#210) for three days (Fig. 2B, lane 3). In both approaches, densitometric analysis of western blots revealed a level of fibrillarin 1.8-fold higher in the absence of functional p53 in cells. This result is in agreement with^27^. In this experiment siRNA-mediated depletion of p53, as assessed by western blotting, was highly efficient (Fig. 2B, see inset).

The nearly doubled level of fibrillarin in cells lacking p53 suggested the possibility that rRNA methylation might be more pronounced under these conditions. This was clearly not the case for any of the 106 positions identified in this work (Table 1 and Supplementary Table S3). Importantly, this demonstrates that doubling the intracellular level of fibrillarin is not alone sufficient to induce hypermodification.

Inhibition of ribosome biogenesis is known to trigger an antitumor surveillance pathway called ‘nucleolar stress’, leading to stabilization of p53 following sequestration of Hdm2 (which normally degrades p53) by unassembled ribosomal components (see refs 35, 39 and 40). This proved also to be the case after siRNA-mediated fibrillarin depletion, as shown by the increased accumulation of p53 detected by western blotting (Fig. 2A). The progressive accumulation of p53 paralleled fibrillarin depletion.

Fibrillarin homologs in budding yeast (where the homolog is called Nop1) and other eukaryotes, including in HeLa cells, have been shown to be important for ribosome biogenesis, and in particular for the initial pre-rRNA processing steps leading to synthesis of the small-ribosomal-subunit rRNA (18S rRNA)^34, 35^. Expecting this to be important in interpreting the results of systematic 2′-O-methylation site mapping after fibrillarin depletion (see below), we examined the extent and kinetics of the impact of fibrillarin depletion on the formation of mature rRNAs in the cell lines used here.

Total RNA was extracted at different time points during fibrillarin depletion (1-, 2-, and 3 days) from both HCT116 p53 +/+ and HCT116 p53 −/− and resolved by denaturing agarose gel electrophoresis. The large mature rRNAs (18S and 28S) were visualized by ethidium bromide staining (Fig. 2C). This revealed that the production of 18S rRNA was indeed severely impacted upon fibrillarin depletion (visible as a 2-fold reduction as early as day 2 of depletion). The accumulation of 28S rRNA was also reduced, especially after 3 days of depletion. As shown in Fig. 2C, however, 28S was always less severely affected than 18S. This suggests that the observed reduction in 28S is likely to be an indirect consequence of inhibition of small ribosomal subunit production (see below). The two siRNAs used (#528, and #529) had largely similar effects.

To gain precise information on the inhibited step(s) of ribosome biogenesis, pre-rRNA processing was analyzed in detail by high-resolution quantitative northern blotting. Specific probes were used to detect all the major known pre-rRNA intermediates (Supplementary Figures 4–5).

Production of three of the four mature rRNAs (5.8S, 18S, and 28S) involves extensive processing of long primary transcripts (47S pre-rRNA) synthesized by RNA polymerase I. In the primary transcripts, the sequences of the mature rRNAs are interspersed with the 5′ and 3′ external transcribed spacers (5′ and 3′ ETS) and the internal transcribed spacers 1 and 2 (ITS1 and 2) (see Supplementary Figure 4).

Fibrillarin depletion led to gradual loss of the initial cleavages at sites 01 and A0 in the 5′ ETS, at site 1 at the 5′ end of 18S rRNA, and at site 2 in ITS1 (see Supplementary Figure 4A). Inhibition of cleavage at sites 01, A0, and 1 was demonstrated by the accumulation of 47S pre-rRNA and 34S RNA (Supplementary Figure 5). The 34S RNA is an aberrant species that arises when the primary transcript is first cleaved in ITS1 in the absence of cleavage in the 5′ ETS (Supplementary Figure 4B). There are at least two alternative pre-rRNA processing pathways in human cells (Supplementary Figure 4, ref. 41). In pathway 1, impairment of cleavage at sites 1 or 2 led to accumulation of 43S or 41S, respectively, while inhibition of cleavage 2 resulted in drastically reduced synthesis of 21S/21S-C and 18S-E (Supplementary Figure 5, panel II), this last species being the immediate precursor of the 18S rRNA. In the second pathway, inhibition at A0 led to a severe reduction in 26S production, and inhibition at A0/1 led to no production of 21S/21S-C or 18S-E (Supplementary Figure 5, panel II). In contrast, accumulation of 32S, a major precursor in the synthesis of the large-subunit rRNAs, was largely unaffected (Supplementary Figure 5, panel III). Consistently, the 12S pre-rRNA, precursor of the 5.8S rRNAs, was also only marginally affected (Supplementary Figure 5, panel III). This indicates that, as suggested above, the observed reduction of 28S is likely an indirect effect. As the processing phenotypes of HCT116+/+ and HCT116−/− proved similar, it appears that the presence or absence of p53 has no significant impact on the involvement of fibrillarin in ribosome biogenesis.

We conclude that fibrillarin depletion primarily affects pre-rRNA processing steps leading to synthesis of the small subunit 18S rRNA, and only slightly impairs production of the large subunit rRNAs. We also conclude that the severity of all the processing phenotypes reported follow the progressive depletion of fibrillarin, indicating these are direct effects.

**Figure 3:**
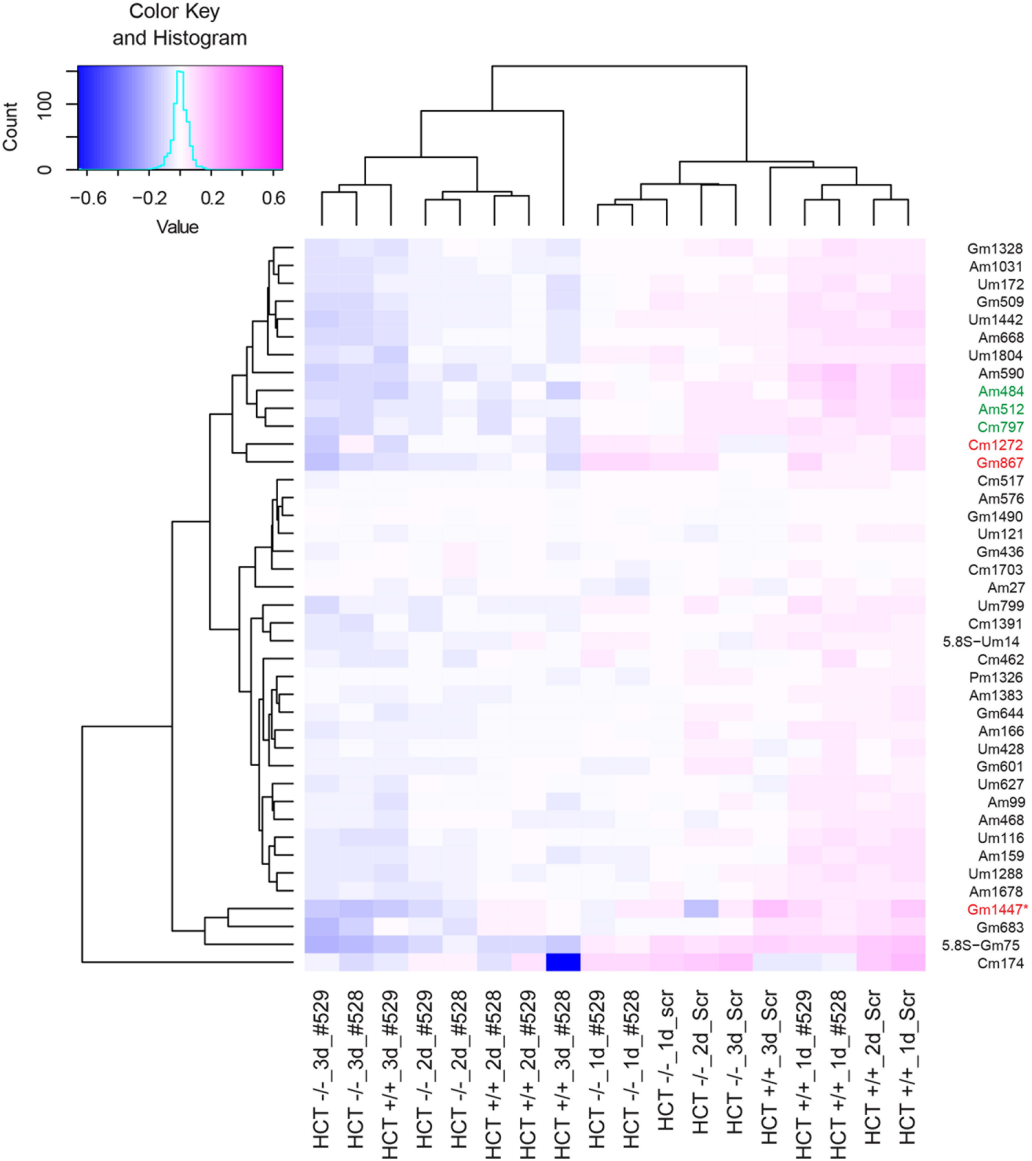
Clustering of 2′-O-methylation on human 18S and 5.8S rRNAs according to the sensitivity of methylation to fibrillarin depletion in the presence or absence of p53 in cells. A methylation score was computed for each position at the different time points of fibrillarin depletion (1, 2, and 3 days) in both HCT116 p53 +/+ and HCT116 p53 −/− cells. The fibrillarin depletion was performed twice independently with two different siRNAs (#528, and #529). The heatmap displays normalized mean methylation scores. All methylation scores were clustered with the software ‘R’ (hclust). The modified positions are indicated on the right. All positions are in 18S rRNA, except two which are in 5.8S rRNA (highlighted as 5.8S-Um14 and 5.8S-Gm75). They are highlighted as follows: asterisk, a position particularly sensitive to fibrillarin depletion; red, hypomodification in HCT116 p53 +/+ and in HCT116 p53 −/− cells; green, hypomodification only in HCT116 p53 −/− cells. The inset shows the color key and histogram.

**Figure 4:**
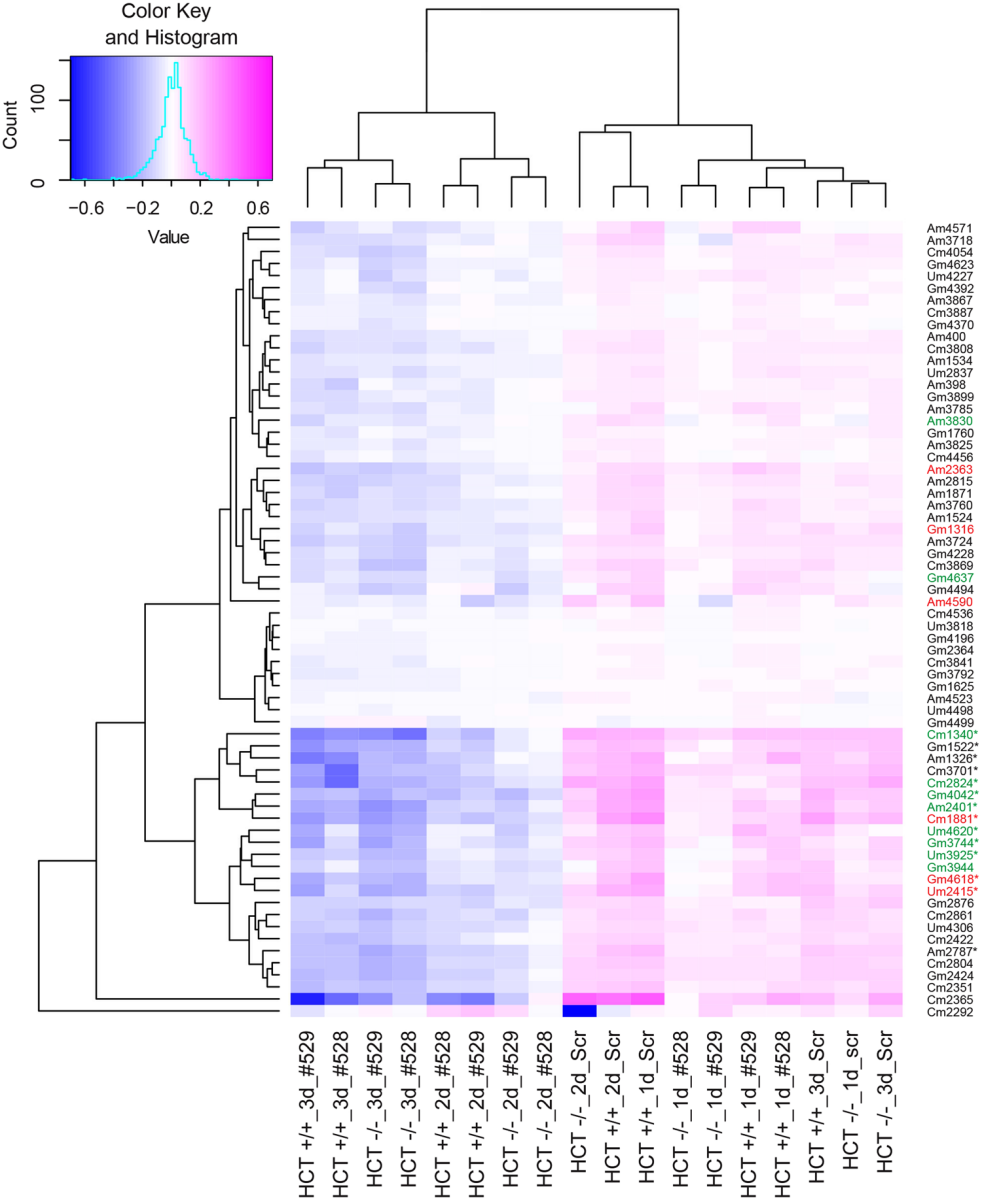
Clustering of human rRNA 2′-O-methylation on 28S rRNA sites according to their sensitivity to fibrillarin depletion in the presence or absence of p53 in cells. Legend as in Fig. 3 for the 2′-O-methylated residues detected on the 28S rRNA. Asterisks, positions particularly sensitive to fibrillarin depletion; red, hypomodification in HCT116 p53 +/+ and in HCT116 p53 −/− cells; green, hypomodification only in HCT116 p53 −/− cells.

RiboMethSeq was performed on total RNA extracted from both HCT116+/+ and HCT116−/− cells at each time point during fibrillarin depletion with each of the above-mentioned siRNAs (#528, and #529). All of the modified positions described above were identified, a methylation score was computed for each, and the methylation scores were clustered with the dedicated software package ‘R’ (Fig. 3 for 18S and 5.8S and Fig. 4 for 28S, see Supplementary Table S3 for complete dataset). The clusters nicely illustrate the consistency of the results obtained with the two siRNAs in each of the two cell lines (Figs 3 and 4). As expected, the methylation levels were found to decrease throughout depletion at nearly all positions (shifting from red to blue in the heatmaps, from right to left, in Figs 3 and 4). Most interestingly, specific positions appeared more affected than others. One site on the 18S rRNA and fourteen on the 28S rRNA appeared particularly sensitive to fibrillarin depletion (labeled with asterisks in Figs 3 and 4 and shown in Table 2) on the basis of the following criteria: on at least two-fold reduction in methylation level by day 3, observed with both siRNAs used (#528 and #529) after a clearly progressive reduction throughout depletion. In Figs 3 and 4, the positions hypomodified in both HCT116 p53 +/+ and HCT116−/− are labelled in red, those hypomodified only in HCT16 p53 −/− are in green. Strikingly, the positions most sensitive to fibrillarin depletion are largely those which are also naturally hypomodified (Figs 3 and 4, Table 2). This is particularly true in the 28S rRNA, where no less than ten of the fourteen positions sensitive to fibrillarin depletion are hypomodified. The mechanisms resulting in partial modification are totally unknown, and this unexpected result indicates that, to some extent at least, hypomodification may simply reflect that the modification machinery has to overcome ‘access constraints’ during subunit biogenesis in order to efficiently reach the substrate residues to be modified.

**Table 2:** Sites of 2′-O-methylation vulnerable to fibrillarin depletion.

	HCT116 p53 +/+	HCT116 p53 −/−	snoRNA guide
**18S**	Gm1447^#^	Gm1447^#^	**SNORD127**
**28S**	Am1326 (1313)	Am1326 (1313)	SNORD18, **SNORD18A**, **SNORD18B**, **SNORD18C**
	Cm1340 (1327)	Cm1340 (1327)^#^	**SNORD104**
	Gm1522 (1509)	Gm1522 (1509)	**SNORD2**
	Cm1881 (1868)^#^	Cm1881 (1868)^#^	**SNORD48**
	Am2401 (2388)	Am2401 (2388)^#^	**SNORD68**
	Um2415 (2402)^#^	Um2415 (2402)^#^	SNORD143, SNORD144
	Am2787 (2774)	Am2787 (2774)	**SNORD99**
	Cm2824 (2811)	Cm2824 (2811)^#^	**SNORD95**
	Cm3701 (3680)	Cm3701 (3680)	SNORD88, **SNORD88A**, SNORD88B, SNORD88C
	Gm3744 (3723)	Gm3744 (3723)^#^	**SNORD87**
	/	Um3925 (3904)^#^	**SNORD52**
	Gm4042 (4020)	Gm4042 (4020)^#^	**SNORD102**
	Gm4618 (4588)^#^	Gm4618 (4588)^#^	**SNORD91A**, **SNORD91B**
	Um4620 (4590)	Um4620 (4590)^#^	**SNORD72**

**Figure 5:**
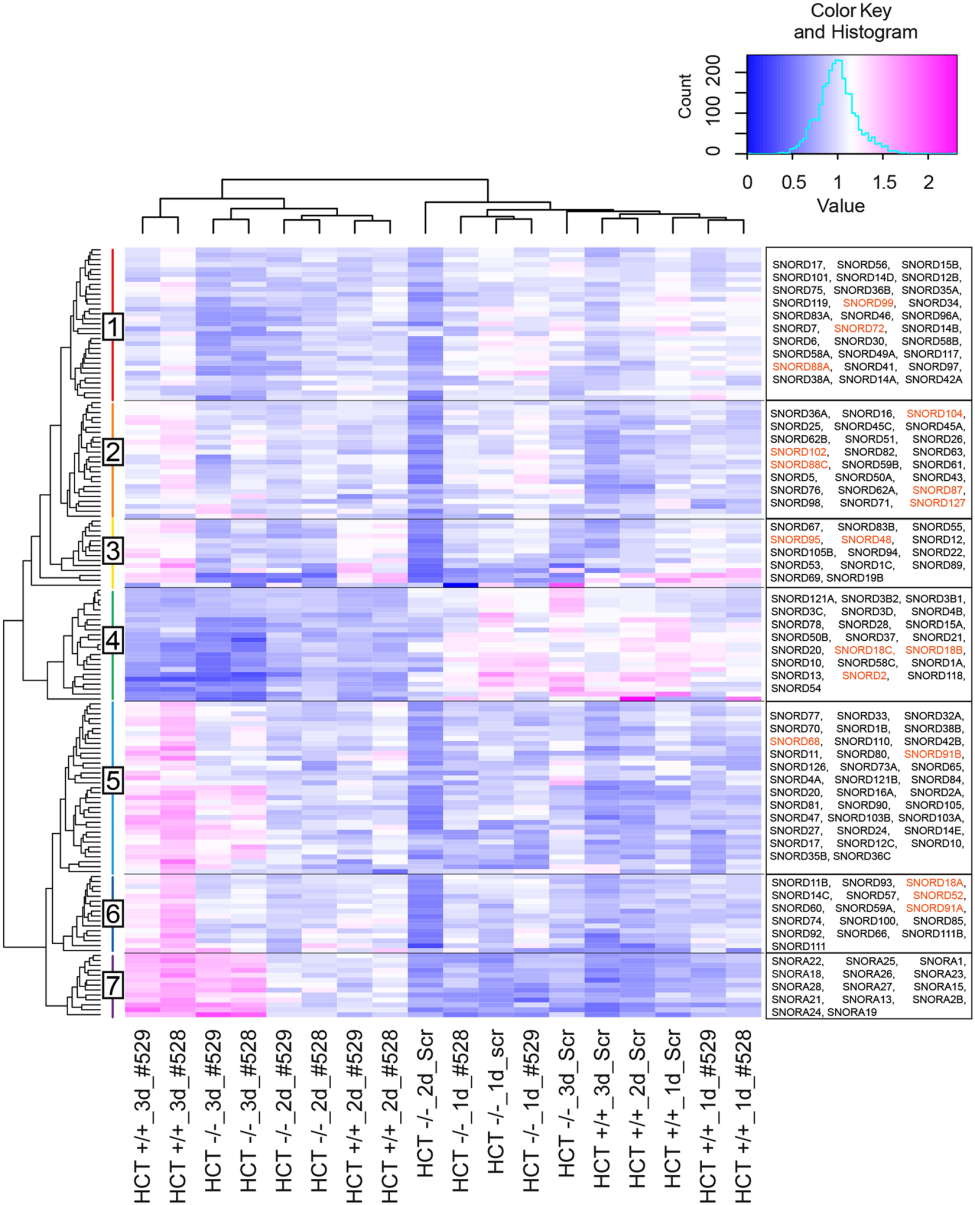
Clustering of snoRNA levels according to their sensitivity to fibrillarin depletion. SnoRNA abundances were inferred from numbers of sequencing reads in the RiboMethSeq dataset and clustered with ‘R’ (hclust). All detected box C/D snoRNAs (SNORD) are represented (categories 1 to 6). Representative H/ACA snoRNAs (SNORA) are shown (category 7). The inset shows the color key and histogram. snoRNAs labelled in orange are involved in the modification of the variable positions identified in this work.

Cut-off: 2-fold reduction after 3 days of depletion, observed with both siRNAs, and after progressive reduction. ^#^Also hypomodified in the indicated cell line (see Table 1). For 28S rRNA: new numbering (old numbering). Putative snoRNA guides for which read counts (intracellular abundances) could be retrieved from the RiboMethSeq dataset (see Fig. 5) are indicated in bold.

At this point we can see how important it was to establish precisely the levels of the various pre-rRNA intermediates over the time course of fibrillarin depletion: it appears that the reduced methylation observed at specific sites cannot be explained solely by reduced production of the precursors harboring them. Most of the positions where methylation appeared particularly sensitive to fibrillarin depletion (fourteen altogether, see Table 2) are on the 28S rRNA, whose main precursor, the 32S, is not particularly affected by depletion (Supplementary Figure 5, panel III). Conversely, we identified only one vulnerable position on the 18S rRNA, whose synthesis was severely affected.

The robustness of the methylation scores after fibrillarin depletion was established in a biological replicate. A side-by-side comparison of the scores computed for each rRNA position after 2 days of fibrillarin depletion mediated by siRNA #529 in two independent cell cultures revealed that the methylation scores are highly correlated (Supplementary Figure 6A) and that the variation in methylation scores is statistically valid (Supplementary Figure 6B).

2′-O-methylation is guided by antisense box C/D snoRNAs whose metabolic stability depends on their efficient assembly with fibrillarin^42, 43^. We thus wondered whether the differentially reduced modification observed at specific positions might be accompanied by differential loss of the corresponding guide snoRNAs.

We established snoRNA abundances over the time course of fibrillarin depletion by retrieving deep sequencing read counts for individual snoRNA species, directly from the RiboMethSeq dataset used to established the methylation scores (Fig. 5). This was possible because we performed RiboMethSeq on total RNA samples. The relative abundances of the snoRNAs detected were normalized to the entire population of ncRNAs (~30,000 species) and were clustered with the software ‘R’ (Fig. 5). The snoRNA counts observed after fibrillarin depletion with either of the two siRNAs used were found to cluster almost identically, highlighting the consistency of the dataset. The robustness of the snoRNA abundance data was investigated with DESeq 2 and the results shown to be highly consistent and statistically valid (Supplementary Figure 7).

The box C/D snoRNAs (SNORD) were grouped in six categories (labeled ‘1 to 6’ in Fig. 5) according to their dependence on fibrillarin to be metabolically stable. As a control, we scored the H/ACA snoRNAs (SNORA), which formed a category of their own (labeled ‘7’ in Fig. 5). None of the H/ACA snoRNAs detected (80 altogether, but only 13 species are shown here for the sake of simplicity) showed a decrease during fibrillarin depletion. This was expected, as these snoRNAs are not known to depend upon fibrillarin for their stability. Actually, they appeared increased in Fig. 5 (see shift from blue to pink with increasing time of depletion in the heatmap), but this simply reflects that reads for the entire population of ncRNA, including box C/D snoRNAs whose levels globally decrease after fibrillarin depletion, were used in our normalization procedure. Upon fibrillarin depletion, C/D snoRNA abundances were either severely reduced (category 4), mildly reduced (categories 1 and 2), or not reduced (categories 3, 5, and 6).

We were able to determine the abundance of 18 of the 23 snoRNAs predicted to be involved in 2′-O-methylation at the positions where this process appeared particularly sensitive to fibrillarin depletion (Table 2). These snoRNAs (labeled in orange in Fig. 5) were found to distribute equally between the six above-described categories of box C/D snoRNAs. There thus appeared no simple direct correlation between the sites whose 2′-O-methylation level is particularly sensitive to fibrillarin depletion and the relative intracellular abundance of the snoRNAs expected to guide them.

As a corollary to our work on 2′-O-methylation, we also identified snoRNAs which appeared particularly resistant to fibrillarin depletion (categories 5 and 6). We predict that in cells, these snoRNAs compete particularly efficiently for fibrillarin binding during snoRNP assembly.

Lastly, we mapped the sites of methylation vulnerability identified in this work to 3-D models of ribosomal subunits, using a specific color code for sites of putative hypomodification, sites sensitive to p53, and sites particularly sensitive to fibrillarin depletion (Fig. 1). Sites of vulnerability turned out not to be located close to functionally important ribosomal sites, such as the DCS or the PTC. On the small subunit, the sites of hypomodification were all found to be located at the subunit periphery, far from the DCS (Fig. 1B). On the large subunit, some sites of vulnerability were found closer to the PTC, but none are in its immediate vicinity (Fig. 1C). In other words, near the DCS and PTC ribose methylation appears robust and not subject to major variations of the type analyzed in this work.

## Discussion

Ribosomal RNA modifications have been shown in multiple cases to optimize ribosome function (e.g. refs 21 and 27). The notion that cells contain a heterogeneous population of ribosomes differing in composition and function and displaying, most notably, different rRNA modification profiles has recently received strong support (discussed in refs 5 and 10). It has also become clear that specialized ribosomes may play important roles in normal processes such as cell differentiation and embryonic development and in diseases such as cancer^8, 9^. This makes it important both to establish the complete repertoire of ribosomal RNA modifications and to identify positions likely to be modified on only a subset of ribosomes, as modification is liable to undergo considerable regulation at these sites.

Here, using the highly sensitive and quantitative deep-sequencing-based technique RiboMethSeq, we have remapped all the sites of ribosomal RNA 2′-O-methylation in two isogenic diploid reference human cell lines, one of which can produce p53 and the other, not. We have identified 106 sites in all: 39 on 18S, 65 on 28S, and 2 on 5.8S (see Fig. 1, Supplementary Figures 1–2, and Table 1).

We have used the term ‘vulnerable’ to qualify the sites which are not modified on all ribosomes at all times. We have identified sites of substoichiometric modification, sites sensitive to the presence of p53 in cells, and sites particularly sensitive to fibrillarin depletion (Tables 1–2). On the basis of statistically validated computed methylation scores (see ref. 15), we report twenty-two putative sites of hypomodification: six on the small subunit and sixteen on the large one. We reveal that most sites of hypomodification are particularly sensitive to the absence of p53 in cells (Table 1). We lastly demonstrate that 2′-O-methylation is more sensitive to progressive depletion of fibrillarin at some specific sites than at others. One site of methylation vulnerability to fibrillarin depletion was identified on the 18S rRNA and fourteen on the 28S rRNA (Table 2).

Interestingly, the few sites of 2′-O-methylation close to the DCS and the numerous ones at the PTC all appear robustly installed in cells, seeming not to undergo the type of variations studied here (Fig. 1). To us, this indicates that these strategically positioned modifications are made constitutively, presumably because they are important in optimizing ribosome function. It suggests that there is likely a ‘core set’ of modifications that do not vary, and that although the theoretical diversity of ribosomes is immense (see Introduction), variability is probably limited to subset of select positions.

Nonetheless, the identification of sites of methylation vulnerability provides ample support to the notion that a wide range of compositionally diverse ribosomes coexist in cells, and that these might have specialized functions in translation. Of direct relevance to disease etiology is the identification of sites whose modification is sensitive to p53, as p53 is mutated in numerous cancers. If the hypomodified ribosomes that accumulate in cells lacking p53 have particular translational capabilities, e. g. if, as recently suggested^44^, they preferentially translate specific mRNA substrates such as those encoding proto-oncogenes, then they might contribute directly to tumorigenesis.

Sites of methylation vulnerability are those most likely to undergo specific regulation during normal processes and in pathophysiological situations. Identifying them provides an initial rational framework for detailed functional characterization, including modeling in cell and animal models. Up to now, functional analysis of the roles played by rRNA 2′-O-methylation has primarily focused on modifications located near functionally important ribosomal sites, such as the PTC or the intersubunit bridges (e.g. refs 16–18 and 20). The putative roles of other, more peripheral, modifications have not been investigated, principally because their abundance makes it very difficult to prioritize them for sophisticated functional analysis (see Fig. 1). Sites of methylation vulnerability constitute a short-list of particular interest in this respect.

Because fibrillarin is required for pre-rRNA processing^35^, we considered it essential to test the possibility that the reduced methylation observed at specific sites might result merely from reduced rRNA production. By establishing the precise impact of fibrillarin on precursor and mature rRNA production in our cells (Fig. 2 and Supplementary Figure 5), we demonstrated that this was clearly not the case over the 3-day depletion period analyzed by RiboMethSeq. Specifically, we showed that the 32S pre-rRNA, i.e. the major precursor of the large subunit rRNAs, is largely unaffected by fibrillarin depletion, although most sites whose methylation is hypersensitive to depletion map to the 28S rRNA, which derives directly from it (Supplementary Figures 4–5). Furthermore, the methylation levels of neighboring residues belonging to the same precursors and the same mature rRNAs are typically affected differentially by fibrillarin depletion (e.g. compare Am576 and Am590, Supplementary Table S3).

An unforeseen conclusion of this work is that the sites whose methylation is hypersensitive to fibrillarin depletion are largely those which are naturally modified only substoichiometrically (Figs 3–4, Table 2). The mechanisms regulating partial modification of specific sites are not known: differential access of the modification machinery to the rRNA substrate residues, influenced by the amounts of functional snoRNPs, is one of many possibilities. Most methylations are expected to occur very early during ribosomal assembly^45^, but several sites may be less accessible than others because of the rapid co-transcriptional folding of the pre-rRNAs. At such sites, decreasing the cell amount of fibrillarin and thus of functional snoRNPs should impair modification. Thus, ‘ready’ access of the modification machinery to the rRNA substrate may limit methylation at certain sites.

Interestingly, we have observed more numerous positions of 2′-O-methylation vulnerability on the large ribosomal subunit than on the small one (Tables 1 and 2). We suggest that this reflects different physical constraints for accessing substrate residues, imposed by the respective architectures of the small and large subunits during ribosomal assembly. If each modification has a certain ‘timeframe’ of completion during subunit assembly, then the small subunit, whose design is highly flexible, may offer additional opportunities for modifications that have ‘missed’ their proper timing, while the large subunit, which is a rigid monolithic block, may be less likely to do so.

Despite the observation that depleting cells of fibrillarin decreases 2′-O-methylation at specific sites, another significant conclusion of our work is that doubling the concentration of fibrillarin in cells is not alone sufficient to increase rRNA methylation levels: cells lacking p53 express twice as much fibrillarin as cells producing p53, but methylation is not increased (Figs 2B, 3 and 4, compare 1d_Scr lanes). In fact, we rather observed the contrary, i.e. specific positions appeared to be less modified in HCT116 p53 −/− cells than in HCT116 p53 +/+ cells (sites referred to as “p53-sensitive”, see Fig. 1, Table 1, Figs 3 and 4, compare 1d_Scr lanes).

Lastly, two recent studies have established the ribosomal RNA methylation landscape of human cells, in a manner quite similar to ours (refs 13 and 32, and see Supplementary Table S1 for an detailed side-by-side comparison of the different studies). While the human rRNA 2′-O-methylation repertoire established by the other two studies is largely similar to ours, neither study investigated the impact of p53 on methylation or identified sites where 2′-O-methylation is particularly vulnerable to fibrillarin depletion.

In conclusion, because the sites of 2′-O-methylation vulnerability identified in this work are likely to undergo specific regulation in normal and pathological situations, we believe they are prime candidates as a focus for future research on ribosomopathies. A list of these sites is now available and directly amenable to functional studies in dedicated cell and animal models.

We suggest that the 106 methylation sites identified here constitute the most common set of ribose methylation sites on human ribosomes (Table 1). It could be, however, that additional sites are modified in particular circumstances such as cell differentiation, embryonic development, or disease.

## Methods

### Cell culture and growth curves

Human cells (HCT116 p53 +/+ and HCT116 p53 −/−) were grown at 37 °C under 5% CO_2_. Growth curves were determined by direct cell counting with a Scepter (EMD Millipore, Billerica, MA, USA). The cell lines used in this work were obtained directly from ATCC and passaged in the laboratory for fewer than 6 months after receipt. All cell lines were diagnosed by ATCC by short tandem repeat (STR) profiling. In HCT116 p53 −/−, the endogenous alleles of p53 were disrupted sequentially by homologous recombination^37^.

### Cell viability count assay

The total number of viable cells and the percentage of viable cells in each sample analyzed were established with a Muse® cell analyser (Merck-Millipore) and the Cell Count and Viability Reagent (REF MCH100102), according to the manufacturers’ instructions.

### RNA interference experiments

HCT116+/+ and HCT116−/− cells were reverse transfected as follows: 1.5 µl of 20 µM DsiRNA (Integrated DNA Technologies) or 20 µM siRNA (Life Technologies) and 4 µl Lipofectamine RNAiMAX (Life Technologies) were mixed with 500 µl Opti-MEM (Life Technologies) in each well of a 6-well plate. After a 20-min incubation at room temperature, 1.5 × 10^5^ cells (for 72 hours), 3 × 10^5^ (for 48 h) 6 × 10^5^ (for 24 h) resuspended in 2.5 ml antibiotic-free medium were seeded into each well. Inactivation was carried out for 24, 48, and 72 hours. The silencers used are listed in Supplementary Table S1.

### RiboMethSeq

Total RNA was extracted as previously described^46^. The NGS-based method of 2′-O-methylation detection was as described for yeast in ref. 15. 100 ng of total RNA was used in our experiments. The Methylation score was calculated as in ref. 14. We systematically used the C score as methylation score (see ref. 15). Briefly, a weighted and normalized average for methylated nucleotide and neighboring positions was calculated and used to evaluate the percentage of methylation.

### Western blotting

Total protein was extracted from ∼1 × 10^6^ HCT116 cells. Cells were washed once with ice-cold 1x phosphate buffered saline (PBS) and lysed in lysis buffer (50 mM Tris-HCl, pH 7.5, 250 mM NaCl, 2 mM EDTA, 0.5% v/v NP-40, 10% v/v glycerol) plus protease inhibitors (complete protease inhibitor, Roche) for 20 min on ice. Lysates were cleared by centrifugation at 20,000 g and 4 °C for 20 min. Protein concentrations were estimated by the Bradford protein assay (Biorad). 30 µg total human protein extract was loaded on a 12% SDS PAGE gel and transferred to a PVDF membrane (GE Healthcare). Antibodies against the following proteins were used at the indicated dilutions: fibrillarin (1:1000 dilution, Abcam); p53 (1:2000 dilution, Santa Cruz; β-actin (1:5000 dilution, Santa Cruz biotechnology). The secondary antibody used was either a horseradish-peroxidase-conjugated anti-mouse IgG (1:5000 dilution, Santa Cruz Biotechnology) or a horseradish-peroxidase-conjugated anti-rabbit IgG (1:5000 dilution, Santa Cruz Biotechnology). Probed membranes were incubated for 10 min with the Super Signal West Pico Chemiluminescent Substrate (Pierce), and the luminescence signal was acquired and processed with a ChemiDoc MP (Bio-Rad).

### Pre-rRNA processing analysis

Total RNA was extracted from HCT116+/+ and HCT116−/− cells in Tri-reagent solution (Life Technologies) according to the manufacturer’s recommendations, except that the RNA pellets were washed with absolute ethanol and then with 75% v/v ethanol. The concentration and quality of the RNA were assessed with a Nano Drop 1000 spectrophotometer (Thermo Scientific). RNA electrophoresis on denaturing agarose gels and northern blotting were conducted as described previously^46^. The membranes were hybridized with ^32^P-labelled oligonucleotides and the signals acquired with a Phosphorimager (FLA-7000, Fuji).

### Bioinformatics

The RiboMethSeq reads were aligned to the reference rDNA sequence NR_046235. Clustering of methylation scores and snoRNA levels was performed with “R” with hclust function using Euclidean distances. To perform statistical analysis of snoRNA abundances after fibrillarin depletion, we used DESeq. 2.

## Electronic supplementary material

Supplementary Information

Supplementary Dataset Number 1

## References

[1] Steitz TA (2008). A structural understanding of the dynamic ribosome machine. Nature reviews. Molecular cell biology.

[2] Sharma S, Lafontaine DLJ (2015). “View From A Bridge”: A New Perspective on Eukaryotic rRNA base Modification. Trends in biochemical sciences.

[3] Polikanov YS, Melnikov SV, Soll D, Steitz TA (2015). Structural insights into the role of rRNA modifications in protein synthesis and ribosome assembly. Nature structural & molecular biology.

[4] Sloan, K. E. *et al*. Tuning the ribosome: the influence of rRNA modification on eukaryotic ribosome biogenesis and function. *RNA biology* Dec 2:1–16. doi:10.1080/15476286.2016.1259781. [Epub ahead of print] (2016). 10.1080/15476286.2016.1259781 PMC5699541 27911188

[5] Lafontaine DLJ (2015). Noncoding RNAs in eukaryotic ribosome synthesis and function. Nature Structural and Molecular Biology.

[6] Danilova N, Gazda HT (2015). Ribosomopathies: how a common root can cause a tree of pathologies. Dis Model Mech.

[7] Yelick PC, Trainor PA (2015). Ribosomopathies: Global process, tissue specific defects. Rare Dis.

[8] Xue S, Barna M (2012). Specialized ribosomes: a new frontier in gene regulation and organismal biology. Nature reviews. Molecular cell biology.

[9] Shi Z, Barna M (2015). Translating the genome in time and space: specialized ribosomes, RNA regulons, and RNA-binding proteins. Annual review of cell and developmental biology.

[10] Dinman JD (2016). Pathways to Specialized Ribosomes: The Brussels Lecture. Journal of molecular biology.

[11] Buchhaupt M (2014). Partial methylation at Am100 in 18S rRNA of baker’s yeast reveals ribosome heterogeneity on the level of eukaryotic rRNA modification. PloS one.

[12] Taoka M (2016). The complete chemical structure of Saccharomyces cerevisiae rRNA: partial pseudouridylation of U2345 in 25S rRNA by snoRNA snR9. Nucleic acids research.

[13] Krogh N (2016). Profiling of 2′-O-Me in human rRNA reveals a subset of fractionally modified positions and provides evidence for ribosome heterogeneity. Nucleic acids research.

[14] Birkedal, U. *et al*. Profiling of ribose methylations in RNA by high-throughput sequencing. *Angew. Chem. Int. Ed*. **53** (2014). 10.1002/anie.201408362 25417815

[15] Marchand, V., Blanloeil-Oillo, F., Helm, M. & Motorin, Y. Illumina-based RiboMethSeq approach for mapping of 2′-O-Me residues in RNA. *Nucleic acids research*, doi:10.1093/nar/gkw547 (2016). 10.1093/nar/gkw547 PMC5027498 27302133

[16] Baxter-Roshek JL, Petrov AN, Dinman JD (2007). Optimization of ribosome structure and function by rRNA base modification. PloS one.

[17] Liang XH, Liu Q, Fournier MJ (2007). rRNA modifications in an intersubunit bridge of the ribosome strongly affect both ribosome biogenesis and activity. Molecular cell.

[18] King TH, Liu B, McCully RR, Fournier MJ (2003). Ribosome structure and activity are altered in cells lacking snoRNPs that form pseudouridines in the peptidyl transferase center. Molecular cell.

[19] Baudin-Baillieu A (2009). Nucleotide modifications in three functionally important regions of the Saccharomyces cerevisiae ribosome affect translation accuracy. Nucleic acids research.

[20] Piekna-Przybylska D, Przybylski P, Baudin-Baillieu A, Rousset JP, Fournier MJ (2008). Ribosome performance is enhanced by a rich cluster of pseudouridines in the A-site finger region of the large subunit. The Journal of biological chemistry.

[21] Jack K (2011). rRNA pseudouridylation defects affect ribosomal ligand binding and translational fidelity from yeast to human cells. Molecular cell.

[22] Motorin Y, Helm M (2011). RNA nucleotide methylation. Wiley interdisciplinary reviews. RNA.

[23] McMahon M, Contreras A, Ruggero D (2015). Small RNAs with big implications: new insights into H/ACA snoRNA function and their role in human disease. Wiley interdisciplinary reviews. RNA.

[24] Bellodi C (2013). H/ACA small RNA dysfunctions in disease reveal key roles for noncoding RNA modifications in hematopoietic stem cell differentiation. Cell reports.

[25] Higa-Nakamine S (2012). Loss of ribosomal RNA modification causes developmental defects in zebrafish. Nucleic acids research.

[26] Yoon A (2006). Impaired control of IRES-mediated translation in X-linked dyskeratosis congenita. Science.

[27] Marcel V (2013). p53 acts as a safeguard of translational control by regulating fibrillarin and rRNA methylation in cancer. Cancer cell.

[28] Schwartz, S. & Motorin, Y. Next-generation sequencing technologies for detection of modified nucleotides in RNAs. *RNA biology*, 0, doi:10.1080/15476286.2016.1251543 (2016). 10.1080/15476286.2016.1251543 PMC5699547 27791472

[29] Gilbert WV, Bell TA, Schaening C (2016). Messenger RNA modifications: Form, distribution, and function. Science.

[30] Limbach, P. A. & Paulines, M. J. Going global: the new era of mapping modifications in RNA. *Wiley interdisciplinary reviews. RNA*, doi:10.1002/wrna.1367 (2016). 10.1002/wrna.1367 PMC5133204 27251302

[31] Jorjani, H. *et al*. An updated human snoRNAome. *Nucleic acids research*, doi:10.1093/nar/gkw386 (2016). 10.1093/nar/gkw386 PMC4914119 27174936

[32] Incarnato, D. *et al*. High-throughput single-base resolution mapping of RNA 2′-O-methylated residues. *Nucleic acids research*, doi:10.1093/nar/gkw810 (2016). 10.1093/nar/gkw810 PMC5388417 28180324

[33] Watkins NJ, Bohnsack MT (2012). The box C/D and H/ACA snoRNPs: key players in the modification, processing and the dynamic folding of ribosomal RNA. Wiley interdisciplinary reviews. RNA.

[34] Tollervey D, Lehtonen H, Carmo-Fonseca M, Hurt EC (1991). The small nucleolar RNP protein NOP1 (fibrillarin) is required for pre-rRNA processing in yeast. The EMBO journal.

[35] Tafforeau L (2013). The complexity of human ribosome biogenesis revealed by systematic nucleolar screening of Pre-rRNA processing factors. Molecular cell.

[36] Tessarz P (2014). Glutamine methylation in histone H2A is an RNA-polymerase-I-dedicated modification. Nature.

[37] Bunz F (1998). Requirement for p53 and p21 to sustain G2 arrest after DNA damage. Science.

[38] Bernier CR (2014). RiboVision suite for visualization and analysis of ribosomes. Faraday Discuss.

[39] Nicolas E (2016). Involvement of human ribosomal proteins in nucleolar structure and p53-dependent nucleolar stress. Nature communications.

[40] Langhendries, J. L., Nicolas, E., Doumont, G., Goldman, S. & Lafontaine, D. L. J. The box C/D snoRNAs U3 and U8 are required for pre-rRNA processing and tumorigenesis *in vitro* and *in vivo*. *Oncotarget*, doi:10.18632/oncotarget.11148 (2016). 10.18632/oncotarget.11148 PMC5312328 27517747

[41] Mullineux ST, Lafontaine DLJ (2012). Mapping the cleavage sites on mammalian pre-rRNAs: where do we stand?. Biochimie.

[42] Watkins NJ (2004). Assembly and maturation of the U3 snoRNP in the nucleoplasm in a large dynamic multiprotein complex. Molecular cell.

[43] Su H (2014). Elevated snoRNA biogenesis is essential in breast cancer. Oncogene.

[44] Marcel, V., Catez, F. & Diaz, J. J. p53, a translational regulator: contribution to its tumour-suppressor activity. *Oncogene*, doi:10.1038/onc.2015.25 (2015). 10.1038/onc.2015.25 25728674

[45] Kos M, Tollervey D (2010). Yeast pre-rRNA processing and modification occur cotranscriptionally. Molecular cell.

[46] Sharma S (2015). Yeast Kre33 and human NAT10 are conserved 18S rRNA cytosine acetyltransferases that modify tRNAs assisted by the adaptor Tan1/THUMPD1. Nucleic acids research.

[47] Khatter H, Myasnikov AG, Natchiar SK, Klaholz BP (2015). Structure of the human 80S ribosome. Nature.

[48] Taoka M (2015). A mass spectrometry-based method for comprehensive quantitative determination of post-transcriptional RNA modifications: the complete chemical structure of Schizosaccharomyces pombe ribosomal RNAs. Nucleic acids research.

