# Supplementary material for: Identification of sites of 2′-O-methylation vulnerability in human ribosomal RNAs by systematic mapping
Source: Sci Rep. 2017 Sep 13;7:11490. doi: 10.1038/s41598-017-09734-9 (PMC5597630; doi:10.1038/s41598-017-09734-9)
Supplement: Supplementary file 1 — Supplementary Information [file 41598_2017_9734_MOESM1_ESM.pdf]

SUPPLEMENTARY INFORMATION

Identification of sites of 2'-O-methylation vulnerability in human ribosomal RNAs by systematic mapping

Sunny Sharma[1,‡], Virginie Marchand[2,‡], Yuri Motorin[2,3*], and Denis L.J. Lafontaine[1,*]

[1]RNA Molecular Biology and Center for Microscopy and Molecular Imaging (CMMI), Fonds National de la Recherche (F.R.S./FNRS) and Université Libre de Bruxelles (ULB), BioPark campus Gosselies, Belgium

[2]Next-Generation Sequencing Core Facility, FR3209 BMCT, CNRS-Lorraine University, 9 avenue de la Forêt de Haye, 54505 Vandoeuvre-les-Nancy, France

[3]IMoPA UMR7365 CNRS-UL, BioPole Lorraine University, 9 avenue de la Forêt de Haye, 54505 Vandoeuvre-les-Nancy, France

[‡] Equal contribution of two first authors

[*]Corresponding authors: yuri.motorin@univ-lorraine.fr , denis.lafontaine@ulb.ac.be

Lead contact: denis.lafontaine@ulb.ac.be

SUPPLEMENTARY FIGURES

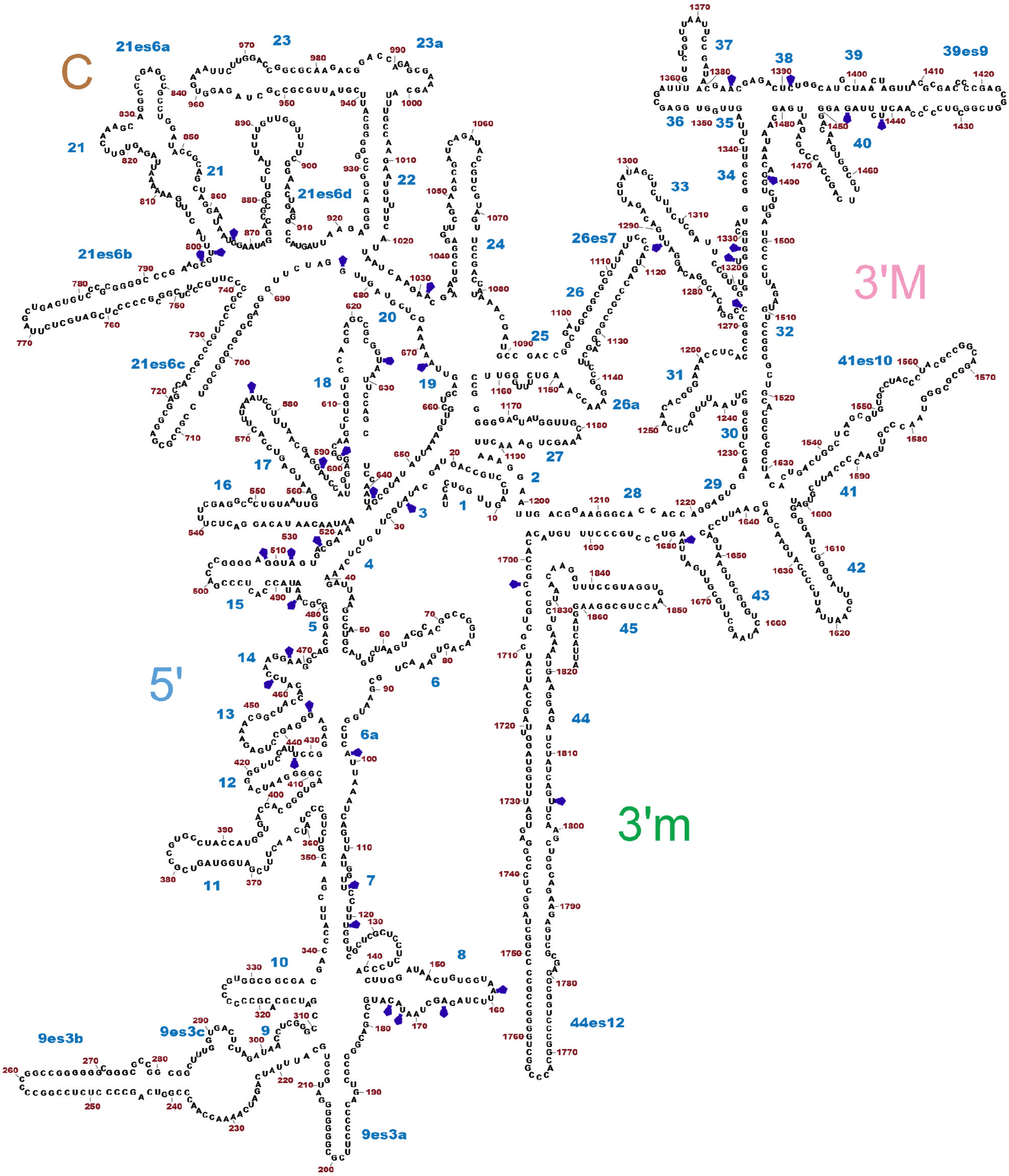

Supplementary Figure 1: Distribution of the 39 2'-O-methylation sites on the secondary structure of the human 18S rRNA

Each 2'-O methylated nucleotide is highlighted with a blue diamond. Structural elements are numbered in cyan (from 1 to 45), with the expansion segments denoted as "es". The nucleotides are numbered (in brown). The four 18S rRNA domains are indicated: 5', central (C), 3' major (3'M), and 3' minor (3'm).

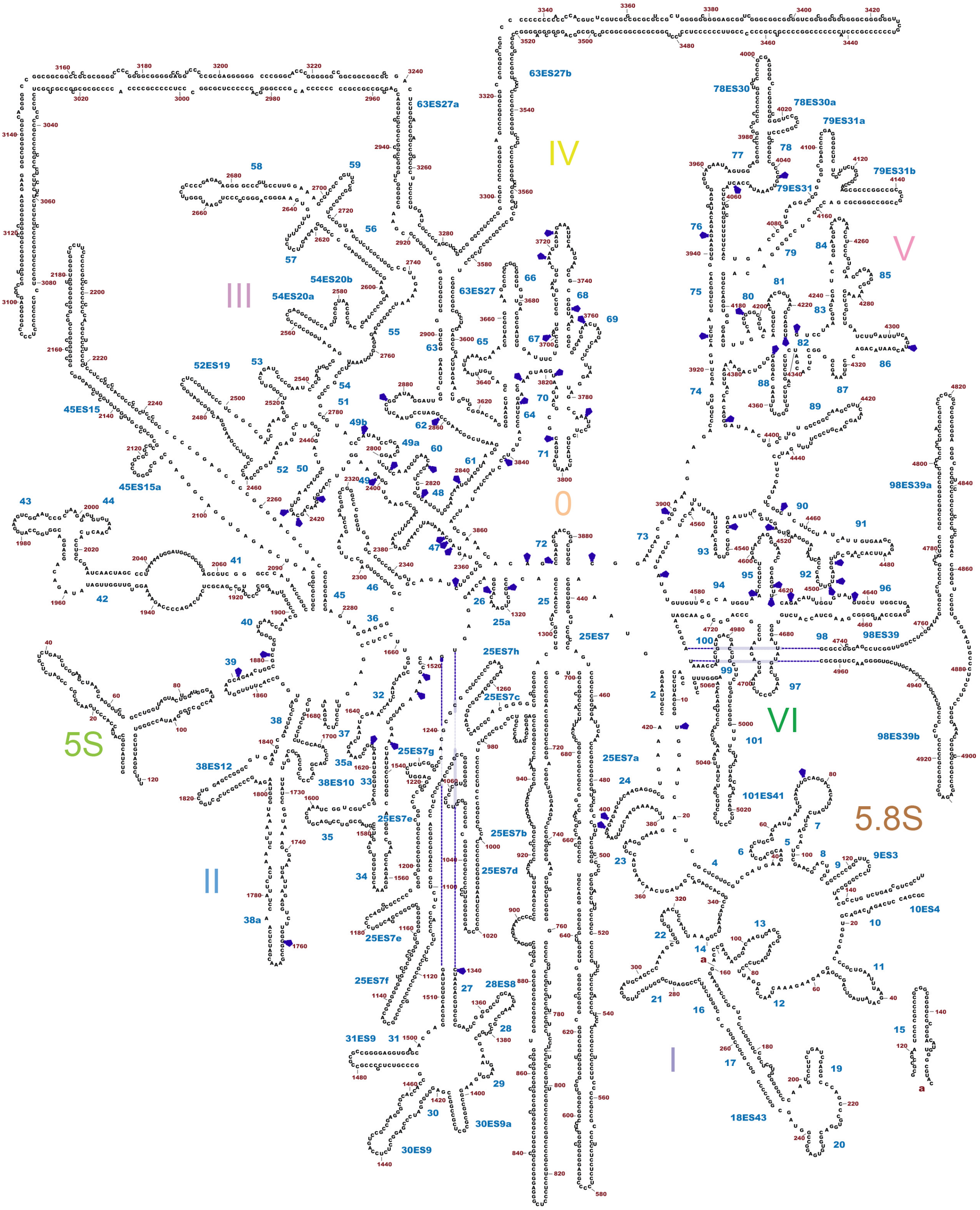

Supplementary Figure 2: Distribution of the 65 2'-O-methylation sites on the secondary structure of the human 28S and of the 2 sites of 2'-O-methylation on 5.8S rRNAs

Legend as in Supplementary Fig 1. Domains I to VI are shown. Expansion segments are denoted as "ES".

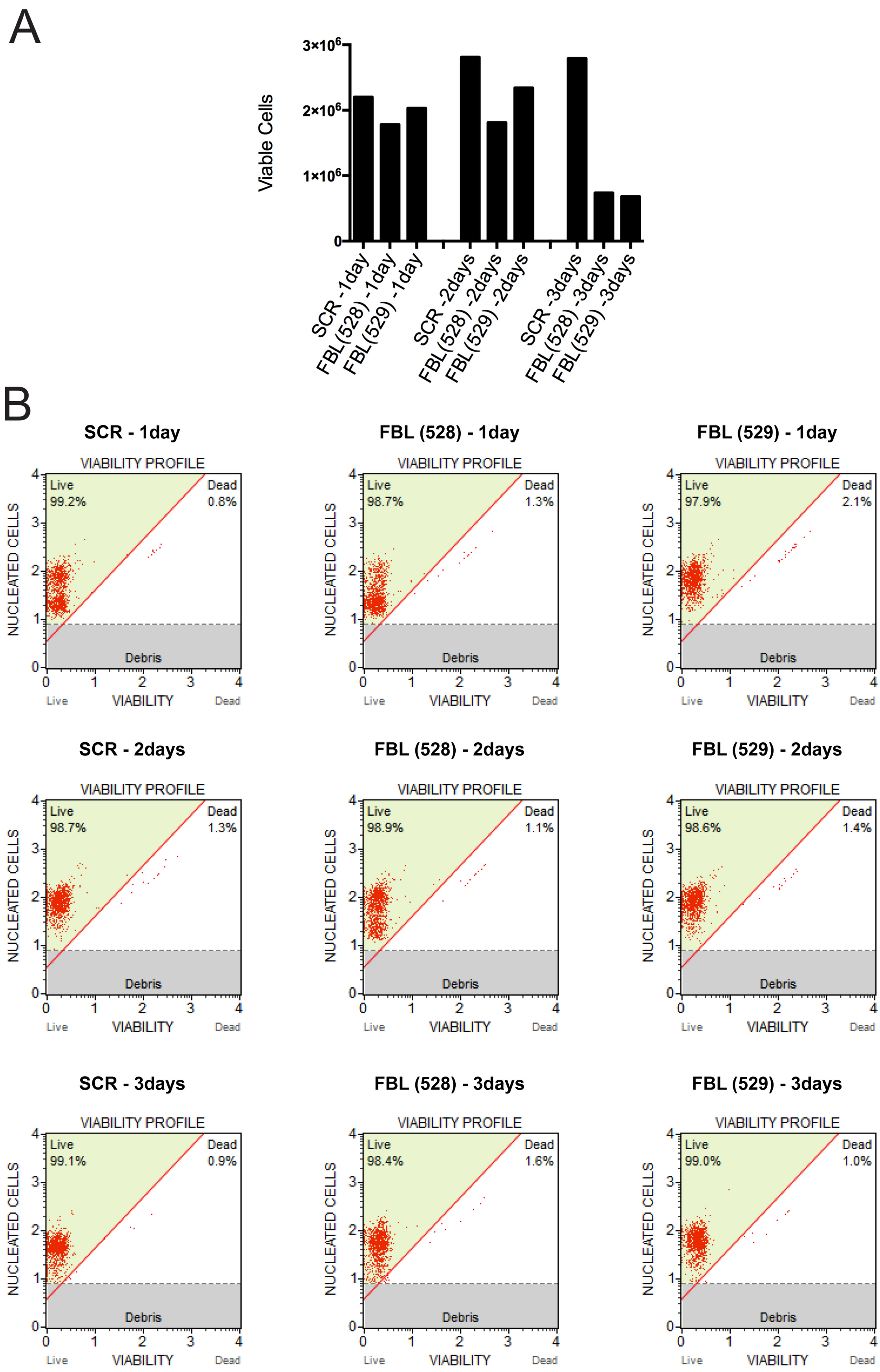
A
Viable Cells
3×10^6
2×10^6
1×10^6
0
SCR -1day
FBL(528) -1day
FBL(529) -1day
SCR -2days
FBL(528) -2days
FBL(529) -2days
SCR -3days
FBL(528) -3days
FBL(529) -3days
B
SCR - 1day
VIABILITY PROFILE
Live 99.2%
Dead 0.8%
FBL (528) - 1day
VIABILITY PROFILE
Live 98.7%
Dead 1.3%
FBL (529) - 1day
VIABILITY PROFILE
Live 97.9%
Dead 2.1%
SCR - 2days
VIABILITY PROFILE
Live 98.7%
Dead 1.3%
FBL (528) - 2days
VIABILITY PROFILE
Live 98.9%
Dead 1.1%
FBL (529) - 2days
VIABILITY PROFILE
Live 98.6%
Dead 1.4%
SCR - 3days
VIABILITY PROFILE
Live 99.1%
Dead 0.9%
FBL (528) - 3days
VIABILITY PROFILE
Live 98.4%
Dead 1.6%
FBL (529) - 3days
VIABILITY PROFILE
Live 99.0%
Dead 1.0%
NUCLEATED CELLS
VIABILITY
Live
Dead
Debris

Supplementary Figure 3: Analysis of cell viability in fibrillarin-depleted cells
The data show that the number of viable cells decreases upon fibrillarin depletion (panel A), in particular after 3 days of depletion. This is as expected, since fibrillarin is essential to small ribosomal subunit biogenesis (Fig 2C and [1]). The data also show that the percentage of viable cells in each sample analyzed remains constant throughout fibrillarin depletion (panel B), ranging from 97.9 to 99.1 %. This is because we only analyzed the adherent cells, which are nearly all viable. In our work, we extracted total protein and total RNA only from viable adherent cells, not from the dying or dead cells present in the cell culture supernatants. All RNA analyses performed in this work were normalized with respect to the amount of total RNA, as established by OD_{260} with a NanoDrop (ThermoFisher).

A, Total number of viable cells in each population.

B, Percentage of viable and dead cells in each sample analyzed. The number and percentage of viable cells in each sample were established by differential fluorescent staining of live and dead cells and fluorescence measurements in a Muse™ cell analyzer. The same number of HCT116 cells was treated for 1, 2, or 3 days with an siRNA against fibrillarin (#528 or #529) or with a control scramble siRNA (SCR) and stained with two selective DNA-binding dyes (one permeates viable cells, providing a 'nucleated cells' index, and the other labels dead and dying cells on the basis of loss of membrane integrity, providing a 'viability' index).

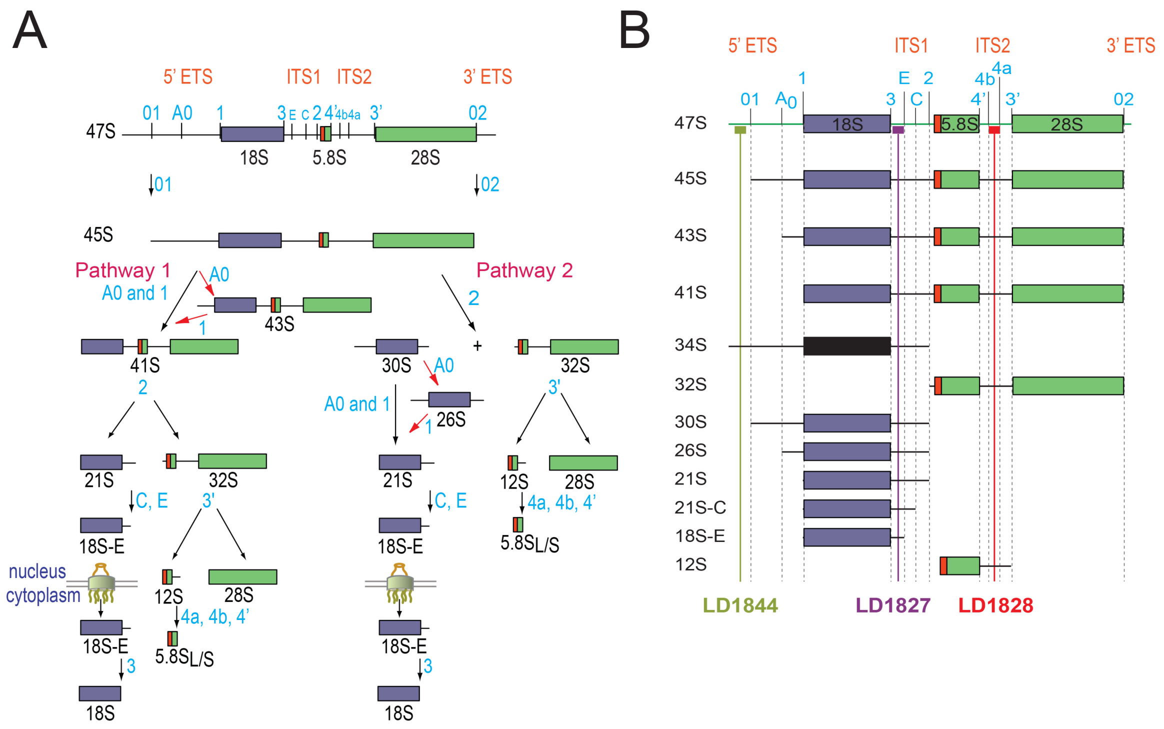

Supplementary Figure 4: Pre-rRNA processing pathways and major pre-rRNA intermediates in human cells

A, Three of the four mature rRNAs, the 18S, 5.8S, and 28S rRNAs are produced from a single RNA Pol I transcript (47S). The 18S rRNA is the RNA component of the small subunit (40S); 5.8S and 28S are incorporated into the large subunit (60S). There is a third rRNA in the 60S subunit, 5S, which is independently produced by RNA Pol III (not shown). The mature sequences are embedded in noncoding 5' and 3' external transcribed spacers (ETS) and internal transcribed spacers (ITS1 and 2). Cleavage sites (in cyan) and alternative pathways are indicated. For details, see Www.RibosomeSynthesis.Com and [2].

B, Northern blot probes used in this work (LD1844, LD1827, and LD1828, see Table S2) highlighting the pre-rRNA species detected.

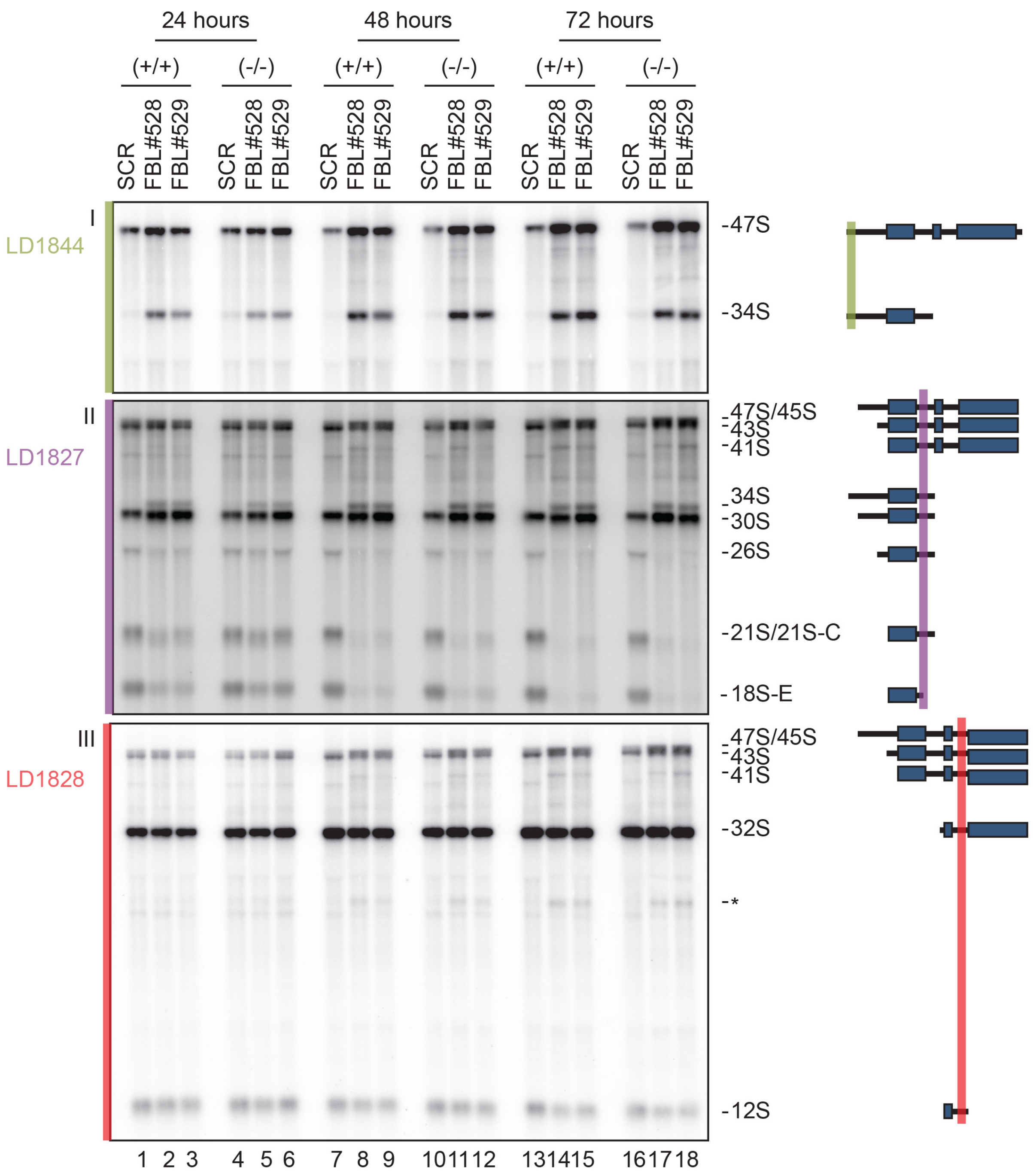

Supplementary Figure 5: Fibrillarin is required for early pre-rRNA processing steps

Total RNA extracted from HCT116 p53 +/+ and HCT116 p53 -/- cells treated with an siRNA specific to fibrillarin (#528 or #529) for 1, 2, or 3 days was resolved on denaturing gels and analyzed by Northern blotting with specific probes (see Supplementary Fig 4). As a control, cells were treated with a non-targeting silencer (SCR). Blots were probed with oligonucleotides LD1844 (panels I), LD1827 (panel II), and LD1828 (panel III). The pre-rRNA species detected are indicated and represented as schematics with the probes used highlighted. A detailed pre-rRNA processing pathway and a description of all the RNA species detected are provided in Supplementary Fig 4. In panel III, a truncated form of 32S (denoted with a star) is detected. It results from activation of cryptic cleavage sites and likely contributes to the minor reduction in the accumulation of the large subunit rRNA.

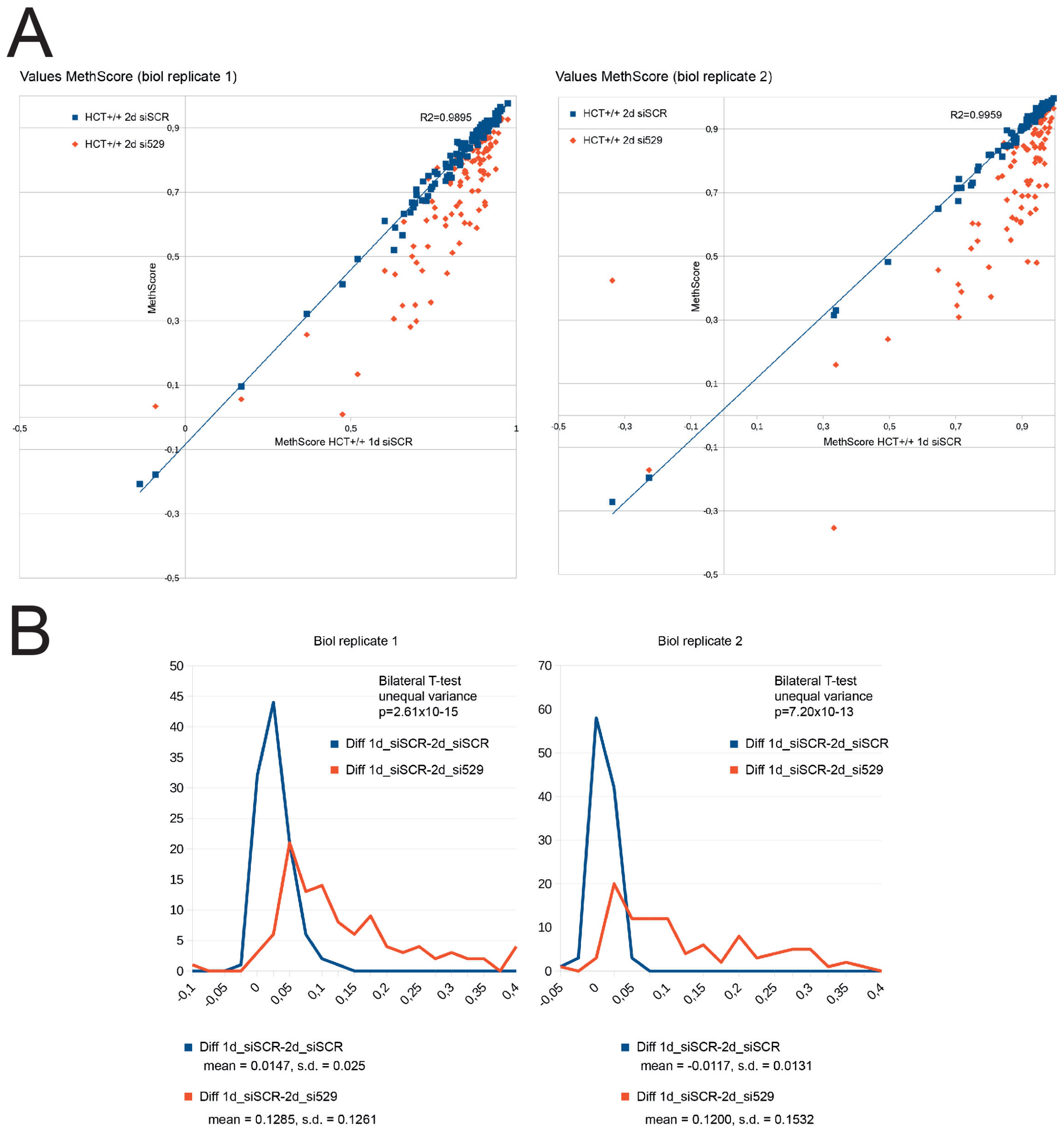

Supplementary Figure 6: Statistical analysis of methylation scores after fibrillarin depletion
Analysis of biological replicates: in order to evaluate the robustness of the methylation scores (MethScores), we repeated our analysis in HCT116 p53 +/+ cells treated with an siRNA specific to FBL (#529) or with the non-targeting scramble control silencer (SCR). The absolute MethScores were somehow higher in the second series of samples (replicate 2), indicating a certain level of biological variability between independent cell cultures. Nevertheless, the observed reductions in 2'-O-methylation scores upon FBL depletion were remarkably consistent.

A, Correlation of MethScores for replicate 1 (left) and replicate 2 (right). Comparison of MethScores in cells treated with the SCR control silencer for 1 or 2 days revealed an excellent correlation (R2>0.985, blue dots and solid blue line). Comparison of MethScores in cells treated for 2 days with the siRNA #529 and MethScores in cells treated with the SCR control

(red dots) revealed a systematic deviation towards lower values, with highly similar patterns in the two independent biological replicates analyzed.

B, Statistical analysis: distribution of MethScore variations in two biological replicates (HCT116 +/+ cells) (same dataset as in panel A). Comparing the MethScores after 1 or 2 days of SCR treatment revealed essentially no differences (in blue, values close to 0 in both biological replicates). A comparison of the MethScores obtained after 2 days of treatment with siRNA #529 and those after treatment with an SCR control revealed similar levels of variation in both replicates (in red). p-values (calculated with a bilateral Student's t-test) revealed the high significance of changes in rRNA 2'-O-methylation (p-value of 2.6x10-15 and 7.2x10-13). Mean and standard deviation (s.d) are indicated for each series.

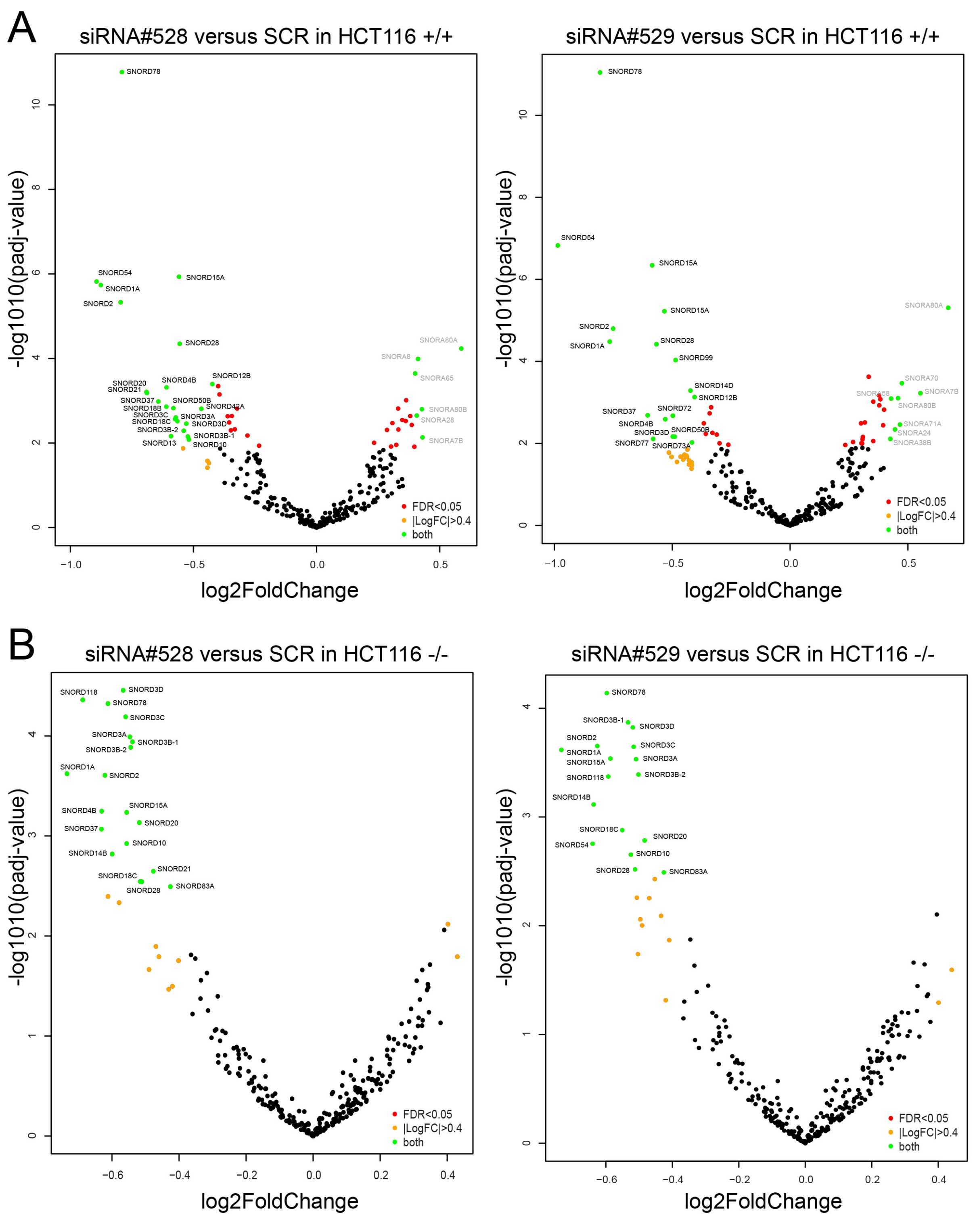

Supplementary Figure 7: Statistical analysis of snoRNA levels after fibrillarin depletion

The volcano plots show that the impact of fibrillarin depletion on snoRNA levels was highly similar, independently of which of two siRNAs (#528 or #529) was used to remove the protein from cells. The data were analyzed with DESeq2, which provides statistical parameters. **A**, HCT116 p53 +/+; **B**, HCT116 p53 -/-. FC, fold change; FDR, false discovery rate; SCR, scramble.

FIG2A:

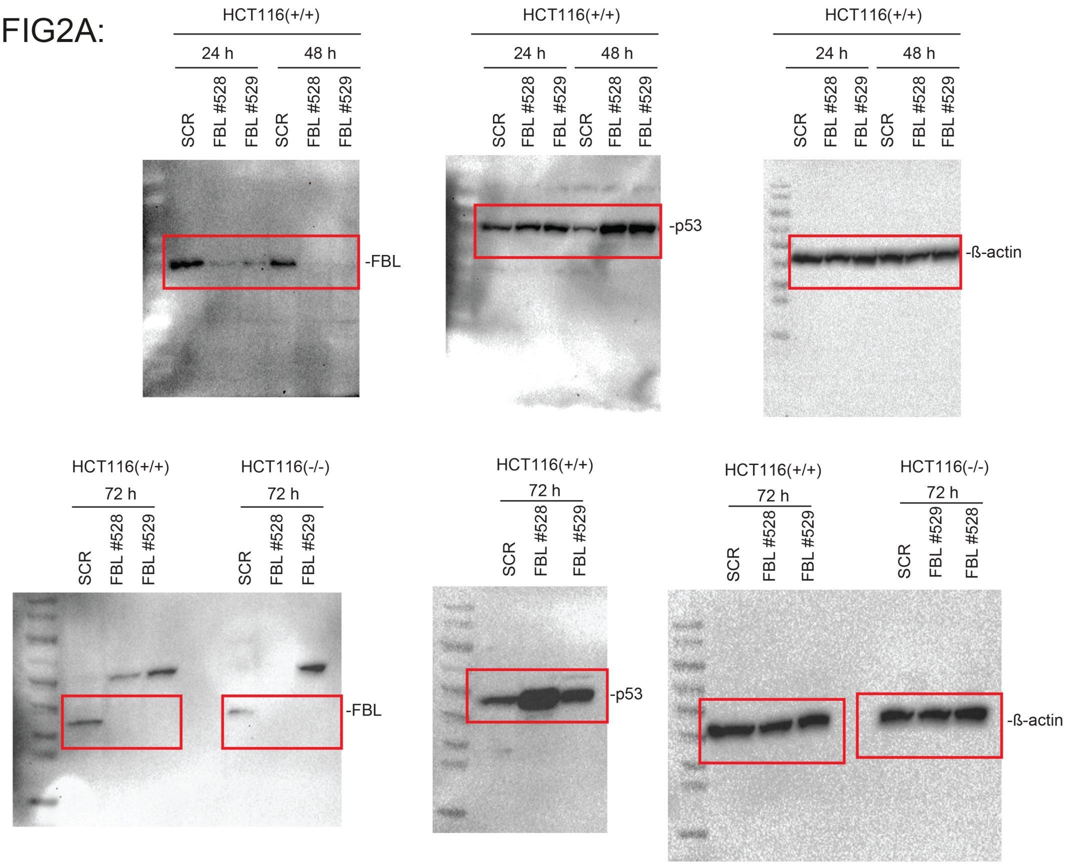

FIG2B:

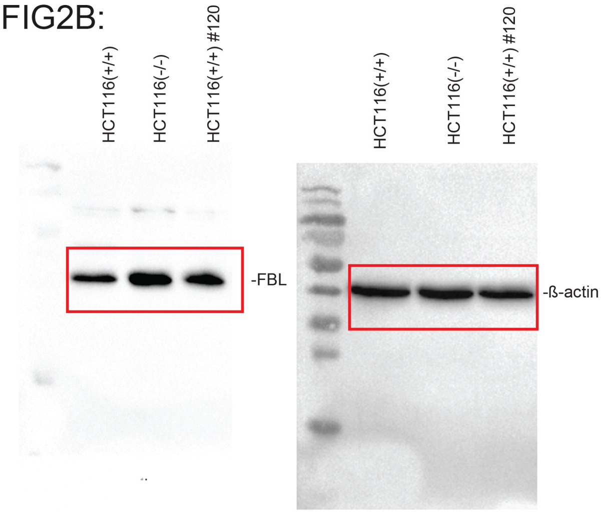

FIG2B (inset):

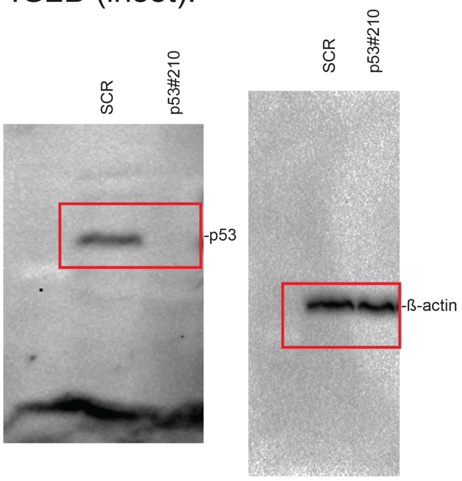

Supplementary Figure 8: Uncropped Western blots, shown in Fig 2A and Fig 2B

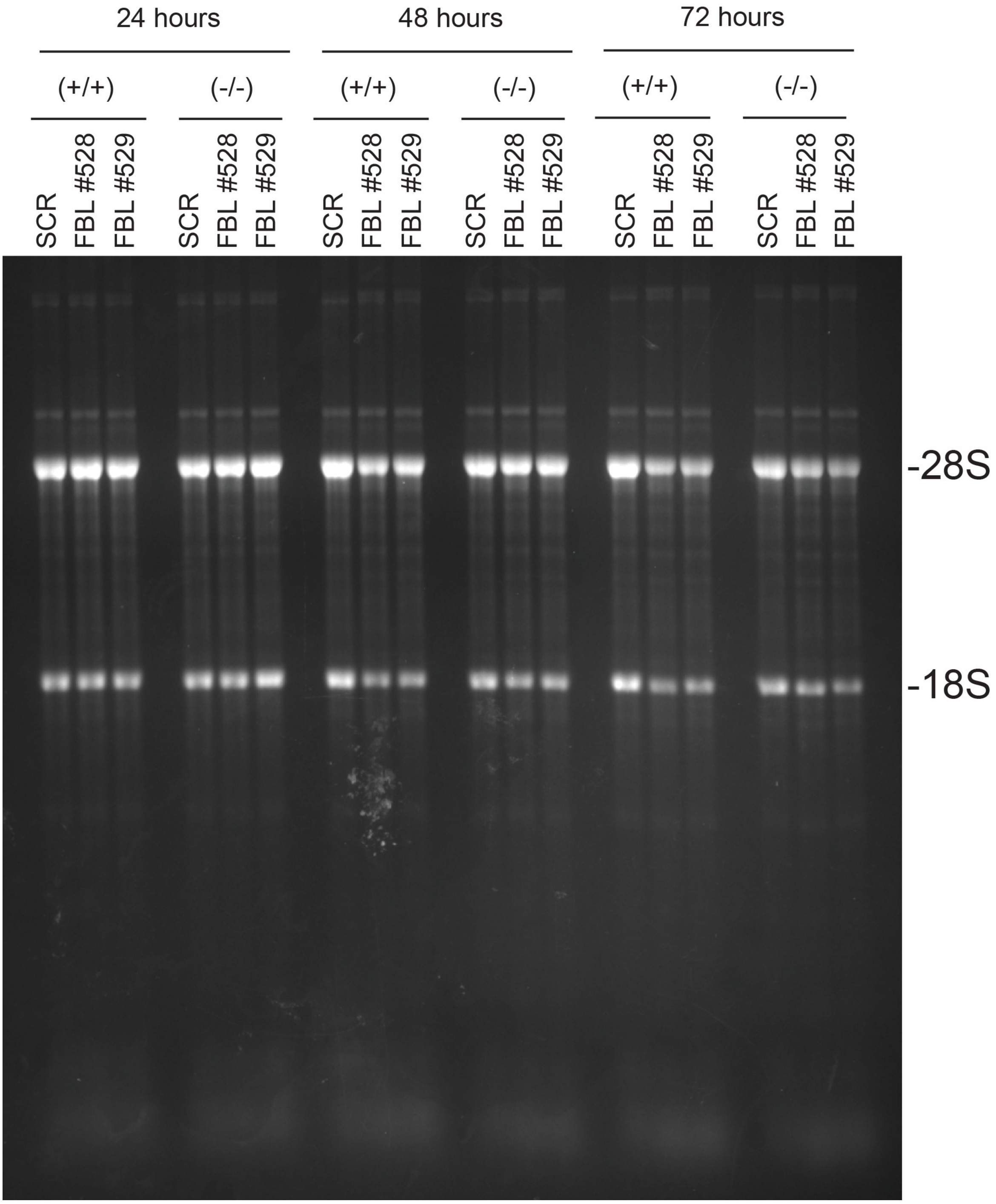

Supplementary Figure 9: Uncropped denaturing agarose gel, shown in Fig 2C

SUPPLEMENTARY TABLES

Supplementary Table S1: Comparison of 2'-O-methylation sites identified on human ribosomal RNAs in different cell lines. A comparison of our work with recent works of others ([3,4]).

18S	Residue type	Our work HCT116 p53 +/+	Our work HCT116 p53 -/-	Nielsen' Lab 2016 HCT116	Nielsen' Lab 2016 HeLa	Oliviero' Lab 2016 HeLa S3
27	Am	0.88	0.83	0.99	1.0	0.767
99	Am	0.83	0.78	0.97	0.98	0.614
116	Um	0.80	0.72	0.92	0.95	0.259
121	Um	0.92	0.89	0.91	0.97	0.238
159	Am	0.87	0.79	0.99	0.98	0.303
166	Am	0.79	0.75	0.99	1.0	n.d.
172	Um	0.83	0.78	0.84	0.96	0.227
174	Cm	0.83	0.78	0.74	0.56	0.064
428	Um	0.8	0.72	0.85	0.88	n.d.
436	Gm	0.89	0.9	0.73	0.58	n.d.
462	Cm	0.85	0.84	0.87	0.85	n.d.
468	Am	0.8	0.76	0.96	0.99	0.329
484	Am	0.73	0.62	0.98	0.98	0.757
509	Gm	0.87	0.86	0.96	0.97	0.282
512	Am	0.75	0.65	0.97	0.94	0.141
517	Cm	0.91	0.89	0.99	0.99	0.674
576	Am	0.95	0.96	0.92	0.89	0.111
590	Am	0.78	0.71	0.89	0.99	0.465
601	Gm	0.81	0.77	0.96	0.99	n.d.
627	Um	0.88	0.86	0.97	0.98	0.495
644	Gm	0.92	0.87	0.93	0.92	0.005
668	Am	0.84	0.79	0.95	0.94	0.029
683	Gm	0.95	0.86	0.96	0.99	0.354
797	Cm	0.73	0.66	0.76	0.85	0.066
799	Um	0.91	0.86	0.97	0.99	0.111
867	Gm	0.64	0.64	0.66	0.73	0.326
1031	Am	0.91	0.88	0.95	0.95	0.608
1272	Cm	0.35	0.32	0.41	0.6	n.d.
1288	Um	0.85	0.8	0.98	0.96	0.105
1326	Ψm	0.84	0.83	0.97	0.99	0.493
1328	Gm	0.84	0.81	0.99	0.99	n.d.
1383	Am	0.93	0.88	0.93	0.95	0.308
1391	Cm	0.9	0.88	0.89	0.98	0.007
1442	Um	0.85	0.79	0.87	0.92	0.100
1447	Gm	0.16	0.07	0.71	0.67	0.031
1490	Gm	0.94	0.94	0.97	0.99	0.026
1678	Am	0.90	0.88	0.98	0.98	0.724
1703	Cm	0.94	0.91	0.94	0.94	n.d.
1804	Um	0.91	0.90	0.83	0.79	0.021
**28S**		Our work HCT116 p53 +/+	Our work HCT116 p53 -/-	Nielsen' Lab 2016 HCT116	Nielsen' Lab 2016 HeLa	Oliviero' Lab 2016 HeLa S3
398 (389)	Am	0.89	0.86	0.98	0.99	n.d.
400 (391)	Am	0.92	0.89	0.97	0.99	n.d.

1316 (1303)	Gm	0.63	0.54	0.68	0.67	n.d.
1326 (1313)	Am	0.79	0.67	0.95	0.97	0.045
1340 (1327)	Cm	0.69	0.64	0.95	0.92	0.011
1522 (1509)	Gm	0.83	0.82	0.96	0.98	0.251
1524 (1511)	Am	0.92	0.83	0.98	0.97	0.263
1534 (1521)	Am	0.94	0.92	0.96	0.97	0.167
1625 (1612)	Gm	0.96	0.95	0.96	0.98	n.d.
1760 (1748)	Gm	0.89	0.87	0.93	0.92	n.d.
1871 (1858)	Am	0.90	0.84	0.97	0.97	0.649
1881 (1868)	Cm	0.47	0.28	0.64	0.63	n.d.
2351 (2338)	Cm	0.86	0.79	0.97	0.97	0.010
2363 (2350)	Am	0.60	0.56	0.97	0.98	0.032
2364 (2351)	Gm	0.91	0.88	0.99	0.99	n.d.
2365 (2352)	Cm	n.d.	n.d.	0.86	0.93	0.026
2401 (2388)	Am	0.69	0.54	0.83	0.71	n.d.
2415 (2402)	Um	0.52	0.36	0.83	0.70	0.359
2422 (2409)	Cm	0.82	0.78	0.9	0.98	0.327
2424 (2411)	Gm	0.91	0.89	0.96	0.99	0.004
2787 (2774)	Am	0.8	0.75	0.92	0.71	0.481
2804 (2791)	Cm	0.9	0.88	0.93	0.89	0.029
2815 (2802)	Am	0.83	0.77	0.91	0.96	0.008
2824 (2811)	Cm	0.68	0.58	0.9	0.94	0.007
2837 (2824)	Um	0.89	0.87	0.99	0.99	0.040
2861 (2848)	Cm	0.83	0.8	0.93	0.85	0.060
2876 (2863)	Gm	0.84	0.82	0.8	0.76	n.d.
3701 (3680)	Cm	0.88	0.82	0.98	0.99	0.100
3718 (3697)	Am	0.88	0.85	0.95	0.94	0.129
3724 (3703)	Am	0.94	0.91	0.99	1.00	0.475
3744 (3723)	Gm	0.71	0.55	0.94	0.9	0.689
3760 (3739)	Am	0.89	0.86	0.97	0.98	0.156
3785 (3764)	Am	0.90	0.82	0.99	1.0	0.063
3792 (3771)	Gm	0.98	0.97	0.99	1.0	n.d.
3808 (3787)	Cm	0.9	0.89	0.95	0.95	0.011
3818 (3797)	Ψm	0.95	0.92	0.91	0.97	0.400
3825 (3804)	Am	0.92	0.88	0.91	0.88	0.690
3830 (3809)	Am	0.75	0.64	0.99	0.99	0.313
3841 (3820)	Cm	0.91	0.9	0.93	0.94	0.061
3867 (3846)	Am	0.82	0.83	0.49	0.64	n.d.
3869 (3848)	Cm	0.87	0.81	0.88	0.84	0.000
3887 (3866)	Cm	0.91	0.89	0.97	0.99	0.182
3899 (3878)	Gm	0.90	0.89	0.94	0.97	0.143
3925 (3904)	Um	0.73	0.61	0.71	0.74	0.038
3944 (3923)	Gm	0.70	0.57	0.8	0.88	0.003
4042 (4020)	Gm	0.74	0.61	0.68	0.59	0.220
4054 (4032)	Cm	0.92	0.89	0.97	0.98	0.270
4196 (4166)	Gm	0.94	0.93	0.96	0.98	n.d.
4227 (4197)	Um	0.88	0.85	0.96	0.96	0.648
4228 (4198)	Gm	0.89	0.86	0.97	0.98	0.025
4306 (4276)	Um	0.79	0.78	0.79	0.92	0.199
4370 (4340)	Gm	0.92	0.91	0.95	0.95	0.026

4392 (4362)	Gm	0.89	0.86	0.99	0.99	n.d.
4456 (4426)	Cm	0.74	0.71	0.95	0.94	0.383
4494 (4464)	Gm	0.90	0.81	0.93	0.96	0.059
4498 (4468)	Um	0.93	0.93	0.98	0.98	0.027
4499 (4469)	Gm	0.75	0.75	0.99	0.99	0.005
4523 (4493)	Am	0.86	0.80	0.99	0.99	0.130
4536 (4506)	Cm	0.95	0.94	0.93	0.89	0.019
4571 (4541)	Am	0.8	0.66	0.87	0.86	0.162
4590 (4560)	Am	0.62	0.51	0.55	0.56	0.055
4618 (4588)	Gm	0.64	0.45	0.78	0.85	0.153
4620 (4590)	Um	0.67	0.57	0.75	0.84	0.153
4623 (4593)	Gm	0.91	0.88	0.89	0.96	0.255
4637 (4607)	Gm	0.68	0.58	0.73	0.80	0.078
5.8S		Our work HCT116 p53 +/+	Our work HCT116 p53 -/-	Nielsen' Lab 2016 HCT116	Nielsen' Lab 2016 HeLa	Oliviero' Lab 2016 HeLa S3
14	Um	0.73	0.71	n.d.	n.d.	n.d.
75	Gm	0.89	0.83	n.d.	n.d.	n.d.

n.d., not determined.

Supplementary Table S2: Synthetic oligonucleotides used in this work

**Northern blot probes**		
**name**	**sequence**	**use**
LD1827	CCTCGCCCTCCGGGCTCCGTTAATGATC	ITS1 probe
LD1828	CTGCGAGGGAACCCCCAGCCGCGCA	ITS2 probe
LD1844	CGGAGGCCCAACCTCTCCGACGACAGGTCGCCAGAGGACAGCGTG	5'-ETS probe
**DsiRNAs** (IDT)		
#528	rUrGrGrUrCrUrCrUrUrUrCrArUrArUrGrGrCrUrCrArArGrGrGrUrC	Fibrillarin depletion
#529	rCrCrUrUrCrCrGrArArArUrCrGrArGrArCrUrCrUrCrUrUrCrUrCrU	Fibrillarin depletion
**siRNA** (Lifetech)		
#210	GAAAUUUGCGUGUGGAGUAtt	p53 depletion

Supplementary Table S3: Primary dataset (see attached Excell spreadsheet)

References

1 Tafforeau, L. *et al.* The complexity of human ribosome biogenesis revealed by systematic nucleolar screening of Pre-rRNA processing factors. *Molecular cell* **51**, 539-551, doi:10.1016/j.molcel.2013.08.011 (2013).

2 Mullineux, S. T. & Lafontaine, D. L. J. Mapping the cleavage sites on mammalian pre-rRNAs: where do we stand? *Biochimie* **94**, 1521-1532, doi:10.1016/j.biochi.2012.02.001 (2012).

3 Krogh, N. *et al.* Profiling of 2'-O-Me in human rRNA reveals a subset of fractionally modified positions and provides evidence for ribosome heterogeneity. *Nucleic acids research*, doi:10.1093/nar/gkw482 (2016).

4 Incarnato, D. *et al.* High-throughput single-base resolution mapping of RNA 2'-O-methylated residues. *Nucleic acids research*, doi:10.1093/nar/gkw810 (2016).
